# Insights into the innate immunome of actiniarians using a comparative genomic approach

**DOI:** 10.1186/s12864-016-3204-2

**Published:** 2016-11-02

**Authors:** Chloé A. van der Burg, Peter J. Prentis, Joachim M. Surm, Ana Pavasovic

**Affiliations:** 1 School of Biomedical Sciences, Faculty of Health, Queensland University of Technology, GPO Box 2434, Brisbane, Qld 4000 Australia; 2 Institute of Health and Biomedical Innovation, Queensland University of Technology, GPO Box 2434, Brisbane, Qld 4000 Australia; 3 School of Earth, Environmental and Biological Sciences, Science and Engineering Faculty, Queensland University of Technology, GPO Box 2434, Brisbane, Qld 4000 Australia; 4 Institute of Future Environments, Queensland University of Technology, GPO Box 2434, Brisbane, Qld 4000 Australia

**Keywords:** Actiniaria, Innate immune system, Transcriptomics, Evolution, TIR domain, NLR, Novel immune genes, Complement cascade, CniFL

## Abstract

**Background:**

Innate immune genes tend to be highly conserved in metazoans, even in early divergent lineages such as Cnidaria (jellyfish, corals, hydroids and sea anemones) and Porifera (sponges). However, constant and diverse selection pressures on the immune system have driven the expansion and diversification of different immune gene families in a lineage-specific manner. To investigate how the innate immune system has evolved in a subset of sea anemone species (Order: Actiniaria), we performed a comprehensive and comparative study using 10 newly sequenced transcriptomes, as well as three publically available transcriptomes, to identify the origins, expansions and contractions of candidate and novel immune gene families.

**Results:**

We characterised five conserved genes and gene families, as well as multiple novel innate immune genes, including the newly recognised putative pattern recognition receptor CniFL. Single copies of *TLR*, *MyD88* and *NF-κB* were found in most species, and several copies of *IL-1R-*like, *NLR* and *CniFL* were found in almost all species. Multiple novel immune genes were identified with domain architectures including the Toll/interleukin-1 receptor (TIR) homology domain, which is well documented as functioning in protein-protein interactions and signal transduction in immune pathways. We hypothesise that these genes may interact as novel proteins in immune pathways of cnidarian species. Novelty in the actiniarian immunome is not restricted to only TIR-domain-containing proteins, as we identify a subset of NLRs which have undergone neofunctionalisation and contain 3–5 N-terminal transmembrane domains, which have so far only been identified in two anthozoan species.

**Conclusions:**

This research has significance in understanding the evolution and origin of the core eumetazoan gene set, including how novel innate immune genes evolve. For example, the evolution of transmembrane domain containing NLRs indicates that these NLRs may be membrane-bound, while all other metazoan and plant NLRs are exclusively cytosolic receptors. This is one example of how species without an adaptive immune system may evolve innovative solutions to detect pathogens or interact with native microbiota. Overall, these results provide an insight into the evolution of the innate immune system, and show that early divergent lineages, such as actiniarians, have a diverse repertoire of conserved and novel innate immune genes.

**Electronic supplementary material:**

The online version of this article (doi:10.1186/s12864-016-3204-2) contains supplementary material, which is available to authorized users.

## Background

The immune system is an ancient and complex system that works to detect and defend against pathogens, as well as to regulate the interaction between microbes and hosts [1–4]. Defence against pathogens in vertebrates is a two-fold mechanism. It consists of the innate immune system, which provides non-specific protection to the host, and the adaptive immune system, which mounts a specific attack against foreign bodies (e.g., microbes) and displays immunological memory [1–4]. Invertebrates, such as cnidarians (corals, jellyfish, hydroids and sea anemones), however, only possess the innate immune system as their primary mode of pathogen defence [5].

Innate immune genes tend to be highly conserved in metazoans, even in early divergent lineages, such as Cnidaria and Porifera [5–9]. However, constant and diverse selection pressures on the immune system have driven the expansion and diversification of different immune gene families in a lineage-specific manner [10–13]. One particular example is the NOD-like receptor family (renamed by [14] as Nucleotide-binding and Leucine-rich Repeat containing gene family (*NLR*)), of which expansions have occurred independently in multiple early divergent lineages [7, 12, 15, 16].

The evolution of immune genes can be complex, as not all functionally conserved immune genes are homologous. Protein domains may evolve independently and subsequently converge on a conserved function or architecture [12, 17]. For example, the functional Toll-like receptor pathway in *Hydra magnipapillata* (freshwater polyp) is formed using a scaffold assembly of TIR-only proteins (Toll/interleukin-1 receptor homology domain) and LRR-only proteins (Leucine-rich repeat) [18–20]. Similarly, it appears that the immunoglobulin-TIR domain combination found in the interleukin receptor family has evolved independently in cnidarians and vertebrates, as these genes share limited sequence similarity [5, 11, 16]. For these reasons, investigating conserved domain architectures can be highly informative in identification and characterisation of the immune repertoire in cnidarians and other early divergent metazoan taxa. Using this approach, the evolution of novel immune genes can also be investigated, through interrogation of novel architectures with domains that have known immune functions. The TIR domain is a well-characterised example; it functions in protein-protein interactions and signal transduction in immune pathways. Such an approach has previously [11] been successfully applied to identify novel immune genes by interrogating TIR-domain-containing architectures.

Like other members of phylum Cnidaria, actiniarians are anatomically simple and develop from only two germ layers (diploblastic). These animals are typically sedentary with no physical protective barriers such as a shell, cuticle or exoskeleton and are therefore directly exposed to the abiotic and biotic conditions surrounding them. Consequently, these organisms have evolved immune defence mechanisms that tend to rely on mucous secretions which consist of antimicrobial peptides, as well as a diverse range of pattern recognition receptors (PRRs) which work in concert with the glycocalyx, to provide a physicochemical barrier [21, 22]. As with other eumetazoans, pathogen recognition in actiniarians is thought to occur primarily via the detection of pathogen associated molecular patterns (PAMPs), using a diverse array of PRRs. Cnidarian immune genes, in particular PRRs, also have a major role in maintaining homeostasis between the host and the beneficial native microbiota, which primarily reside on the epithelium [19, 23], although many cnidarians also undergo endosymbiosis with dinoflagellates [24].

Current genomic resources for cnidarians have been limited to a few key model species, including *Nematostella vectensis* (starlet sea anemone) [6]*, Acropora digitifera* (coral) [25], *H. magnipapillata*. [26] and *Aiptasia* sp. (sea anemone) [27]. Interrogation of these genomic resources has revealed that the cnidarian genome is surprisingly complex. In fact, cnidarians have maintained both eumetazoan and early divergent metazoan gene families, some of which have been lost in invertebrate models *Drosophila melanogaster* and *Caenorhabditis elegans* [5, 9, 28–30]. In particular, the genome of the sea anemone *Nematostella*, has been shown to be more similar to vertebrates than other invertebrate model species, with gross genomic structure and exon/intron arrangement similar to vertebrates [6]. While these few cnidarian species have been crucial in elucidating the complexity of the cnidarian genome, in this highly speciose phylum, more resources are needed to facilitate further in-depth comparative evolutionary and phylogenetic analyses.

Actiniarians are uniquely suited for the study of the origin and evolution of the innate immune system. This is principally attributed to their phylogenetic placement as members of Cnidaria, the sister phylum to Bilateria. Their simple anatomical features and complex, vertebrate-like genomes make Actiniarians an interesting candidate for understanding how novel innate immune genes evolve within eumetazoan species that have limited physical defences. Furthermore, using Actiniarians as a model for understanding anthozoan immunity will help provide insights into other issues facing anthozoan species, such as coral bleaching and disease. To better understand the evolution of the innate immune system, here we performed a comprehensive and comparative investigation of the actiniarian innate immune gene repertoire with the aim to provide preliminary insights into the key processes that have shaped the cnidarian immunome. Specifically, we have examined gene family evolution of candidate immune genes, to better understand the role of gene gain and loss events, the generation of new genes and the acquisition of genes from other species, as key drivers of the innate immune gene repertoire in actiniarians.

## Results

### Sequencing, assembly and annotation

Whole organism extractions of total RNA were performed from *Actinia tenebrosa* (red, brown, green and blue colourmorphs, designated 1, 2, 3 and 4 respectively) *Anthopleura buddemeieri, Aulactinia veratra, Calliactis polypus* (designated 1 and 2)*, Telmatactis* sp. and *Nemanthus annamensis*. Sequencing was performed on an Illumina NextSeq 500, and raw reads per sample ranged between 51,620,970 and 220,632,160. These reads were assembled into 116,120–212,774 contiguous sequences (contigs) for each transcriptome. All raw reads were submitted to the NCBI Sequence Read Archive (SRA) under one BioProject (PRJNA313244); accession numbers for individual transcriptomes can be viewed in Additional file 1: Table S1. Assembly statistics as well as completeness scores can be found in Additional file 1: Tables S2 and S3, which also includes assembly metrics for raw reads downloaded from the SRA for *Aiptasia pallida, Anthopleura elegantissima* and *Nematostella vectensis*. A total of 13 transcriptomes were used in this study, from 9 actiniarian species.

### Gene ontology

Gene ontology (GO) terms were assigned to between 21,750 and 43,791 transcripts using Trinotate (Additional file 1: Table S4). WEGO analysis indicated a similar distribution of GO terms among assemblies (Additional file 1: Figure S1). CateGOrizer analysis showed that the number of genes assigned immune class GOs ranged from 7239 to 12,515 across species, with the exception of *A. pallida* which had only 1500 genes (Additional file 1: Table S5 and Figure S2). Stress response and protein metabolism were the most frequently assigned immune class GO terms for all species, while the ‘immunology, immune response’ term corresponded to approximately 2.7 % of the total number of genes in this GO category, except *A. pallida* which had 0.5 %. The results from CateGOrizer showed that most assemblies have a similar distribution of GO terms associated with the immune class. *A. pallida* showed the most variation from the other transcriptomes, with a much lower fraction of genes in all categories except categories relating to metabolism (i.e., catabolism and protein, lipid and carbohydrate metabolism).

### RSEM

RNA-Seq by Expectation-Maximization (RSEM) analysis of transcriptomes was performed to verify candidate transcripts are expressed and as a guide for choosing the most highly expressed candidate genes for PCR validation i.e., transcripts with the highest FPKM (Fragments Per Kilobase of transcript per Million mapped reads) score were chosen in most instances for PCR validation. The most highly expressed immune transcripts in all transcriptomes for which RSEM was performed (*A. tenebrosa* (1), *A. buddemeieri*, *A. veratra, C. polypus* (2)) were all NLR transcripts, with FPKMs of 84.69 for *A. tenebrosa*, 22.76 for *A. buddemeieri*, 65.74 for *A. veratra* and 370.7 for *C. polypus.*

### Candidate gene counts and architecture

Candidate gene analysis revealed that for most species, a single gene copy of *TLR*, *MyD88* and *NF-κB* was identified (Table 1), with very few species having multiple isoforms of these genes (Additional file 2: Table S6). In contrast, multiple copies of *NLR* and *IL-1R-*like were found in all species. Complete copies of *NLR* genes were found in most species (with the exception of *A. elegantissima* and *Telmatactis* sp., where only partial sequences were found) ranging from 2 to 4 complete and 1–8 partial copies of *NLR* genes, with total (complete and partial) gene copy numbers ranging from 3 to 10 (Table 1 and Additional file 2: Table S7). Complete copies of *IL-1R-*like genes were found in all species, ranging from 1 to 7 complete gene copies and 3–7 total gene copies when considering partial sequences as well (Table 1 and Additional file 2: Table S7).

**Table 1:** Candidate gene counts in each transcriptome

Species	*TLR*	*MyD88*	*NF-κB*	*NLR*	*IL-1R-*like
*A. tenebrosa* (1)	1	1	1	2	3
*A. tenebrosa* (2)	1	1	1	2	7
*A. tenebrosa* (3)	1	1	1	2	4
*A. tenebrosa* (4)	1	1	1	4	3
*A. pallida*	-ǂ	1	0*	2	2
*A. buddemeieri*	1	1	1	3	2
*A. elegantissima*	1	1	1	0†	3
*A. veratra*	1	1	1	2	4
*C. polypus* (1)	0	1	1	2	3
*C. polypus* (2)	1	1	1	4	5
*N. annamensis*	1	1	1	2	4
*N. vectensis*	1	1	0*	3	2
*Telmatactis* sp.	0	0	1	0†	1

Counts are conservative, i.e., they refer to the number of genes with a complete ORF only, not isoforms or partial genes found in each transcriptome (see Additional file 2: Tables S6 and S7 for gene, isoform and partial gene counts). Genes were considered complete when a full open reading frame was found (start and stop codons) and contained the canonical Pfam domains (see Methods). Shown are the Toll-like receptor (*TLR*), Nucleotide-binding and Leucine-rich Repeat containing gene family (*NLR*), Myeloid Differentiation primary response gene 88 (*MyD88*) and Nuclear Factor kappa-light-chain-enhancer of activated B cells (*NF-κB*) and Interleukin-1 receptor (*IL-1R-*like). ǂNo TLR in *Aiptasia* genome [27]. *NF-κB in *Nematostella* does not have ankyrin repeats [71]; here NF-κB was only considered complete if it contained the RHD and ankyrin repeats (see Methods). *Aiptasia also has only a RHD-IPT (no ankyrin repeats) containing *NF-κB* (AIPGENE8848 sp|P19838|NFKB1). †Partial genes found, see Additional file 2: Table S7

**Fig. 1:**
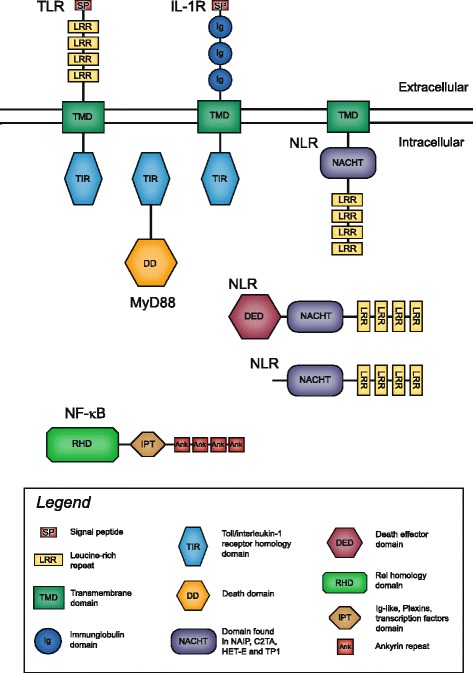
Typical protein domain architecture for the five candidate genes and their hypothesised cellular location. Structures depicted show typical architecture; other variations exist. Depictions based on Pfam annotations and SMART visualisation. Shown are: TLR, Toll-like receptor; NLR, Nucleotide-binding domain and Leucine-rich Repeat containing gene family; MyD88, Myeloid Differentiation primary response gene 88; NF-κB, Nuclear Factor kappa-light-chain-enhancer of activated B cells; IL-1R-like, Interleukin-1 receptor. Depictions of ankyrin repeats, LRRs, TMD and Igs do not reflect the exact number in each gene

All TLR and IL-1R-like predicted peptides had transmembrane domains (TMDs) and all MyD88 and NF-κB predicted peptides did not. Transmembrane domain analysis of NLRs, however, revealed 8 out of 35 total (including complete and partial) NLRs had TMDs. Multiple (3–5) TMDs were identified in TMD containing NLRs and were clustered at the N-terminus. TMD containing NLRs were found in *A. pallida*, *A. tenebrosa*, *A. buddemeieri* and *C. polypus*. The most common protein domain architecture of all candidate genes and their hypothesised location in the cell are shown in Fig. 1.

### Candidate and novel gene validation

To validate candidate and novel genes, PCR amplification from *A. tenebrosa*, *A. buddemeieri, A. veratra* and *C. polypus* was performed. PCR products were sequenced and realigned to the contig from which primers were designed for each transcript. Sequences were considered complete when they aligned to the transcript with at least 94 % pairwise sequence similarity to the ORF, following trimming. A total of 15 complete and 3 partial sequences were validated, with at least one representative sequence from each of the 5 candidate genes, and one representative sequence from each of the 3 novel genes. All validated sequences were submitted to GenBank® (NCBI) and accession numbers can be viewed in Additional file 3: Table S14.

### Presence/absence of other innate immune genes

Additional innate immune genes were investigated in this study for their presence/absence in actiniarian species. These included key receptors and proteins in the complement pathway (i.e., CniFL, which is hypothesised to function in the lectin-complement pathway and complement proteins MASP, C3, Factor B, C6 and Factor I), as well as genes encoding protein domains known to have immune function (i.e., SRCR and C-type Lectin domains). The results are presented in Table 2.

**Table 2:** Gene counts and presence/absence of other innate immune genes

Species	CniFL	MASP	SRCR	C-type Lectin domains	C3 family	Factor B (Bf) family	C6 family	Factor I (If) family
*A. tenebrosa* (1)	5*	1	114	83	+	+	-	-
*A. tenebrosa* (2)	7	1	131	89	+	+	-	-
*A. tenebrosa* (3)	2	1	121	76	+	+	-	-
*A. tenebrosa* (4)	2	1	105	70	+	+	-	-
*A. pallida*	4	1	51	87	+	+	-	-
*A. buddemeieri*	2*	1	135	88	+	+	-	-
*A. elegantissima*	0	1	60	57	+	+	-	-
*A. veratra*	5	1	129	111	+	+	-	-
*C. polypus* (1)	6	1	61	65	+	+	-	-
*C. polypus* (2)	5	1	74	84	+	+	-	-
*N. annamensis*	3	1	39	57	+	+	-	-
*N. vectensis*	1	1	39	67	+	+	-	-
*Telmatactis* sp.	2	1	59	41	+	+	-	-

Gene counts shown are non-conservative, i.e., they refer to the number of contigs identified by Trinity as different genes, and may include contigs with partial ORFs. Only complete ORFs used in subsequent analysis for CniFL genes. *PCR validated. + present; - absent. Abbreviations are as follows: CniFL, Cnidarian Ficolin-like protein; SRCR, Scavenger-Receptor Cysteine Rich; MASP, Mannan-binding lectin (or mannose-associated) Serine Protease 1

### Identification of novel immune genes

**Fig. 2:**
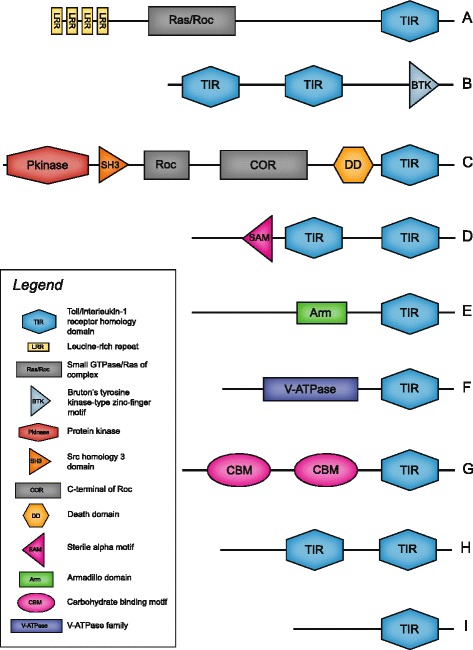
Graphical representation of the novel TIR domain containing immune gene repertoire. Typical architectures are shown; other variations do exist. Architectures were designed from SMART visualisation and Pfam annotations. Depicted lengths of proteins and domain position are informed estimates, with the longest protein (**c**) at about 1,300 aa. Subtypes of domains are not shown for alternate annotations of TIR (e.g. TIR_2 or TIR_like), Pkinase, SH3, LRR, SAM or CBM domains. TIR-only proteins (**h** and **i**) often included a ‘DUF1863’ domain, described as a TIR-like domain, in place of TIR. **a**-**c**: Novel Genes 1, 2 and 3 respectively; these are described in more detail in text. **d**-**i**: Additional novel genes also identified. See Additionalfile 2: Table S9 for full list of novel genes across all species

Taxonomically-restricted transcripts with novel domain architectures were identified in all species, but not all novel transcripts were found in all species examined (Additional file 2: Tables S8–S10). Novel genes with potential immune function were identified based on novel architectures that included the TIR (or TIR_2) domain. A representation of the full repertoire of these genes is shown in Fig. 2. Three of the most common transcripts were investigated further and designated as Novel Genes 1, 2 and 3.

Novel Gene 1 (NG1) was the most commonly observed transcript with novel domain architecture and was found in all species examined. NG1 includes the Pfam domains Leucine-rich repeat (LRR), Miro-like protein (Miro), Ras family (Ras) and Toll/Interleukin-1 receptor homology domain (TIR_2). SMART domain annotation represented the Miro and Ras domains as either Ras family, Ras of complex (Roc) or small GTPase, all of which are GTPase family domains. A low threshold C-terminal of Roc (COR) domain was also found ~300 aa downstream in all genes, which may be essential to the function of the GTPase domain and is always found downstream of Ras [31]. NG1 is represented in Fig. 2a.

BLASTx analysis of NG1 against the TrEMBL database (Additional file 2: Table S9) identified the only significant hit as a *Nematostella* predicted protein. Swiss-Prot BLASTx searches (Additional file 2: Table S9) identified this gene as an F-box/LRR (FBXL) protein from multiple vertebrate species. The FBXL protein, however, only has the LRR region in common with NG1, indicating there is no ortholog of NG1 in vertebrates. Further investigation of this gene with known TIR and TIR_2 architectures from the EMBL-EBI [32] domain database identified 362 unique architectures with the TIR_2 domain (PF13676) and 381 unique architectures with the TIR domain (PF01582), but the TIR domain was not found in combination with any GTPase (Roc, Ras or COR). The TIR_2 domain was identified in combination with one or more of the GTPase domains in multiple bacteria species, including cyanobacteria, filamentous bacteria, purple sulfur bacteria and flavobacteria, and with an additional C2 domain in the pacific oyster, *Crassostrea gigas* (C2 domain – targets proteins to cell membranes), and a MBT domain in the placozoan, *Trichoplax adhaerens* (MBT – repeat of unknown function). Table S10 (Additional file 2) provides details of the variable domain architectures found that could represent orthologous genes to NG1.

Novel Gene 2 (NG2) was identified in six species, *A. tenebrosa* (2, 3 and 4)*, A. pallida, A. elegantissima, C. polypus* (2), *N. annamensis* and *N. vectensis*, and included one or two TIR_2 domains upstream of a BTK motif (Bruton's tyrosine kinase-type zinc-finger motif). Only a single predicted *Nematostella* protein was identified in TrEMBL and no proteins were identified in Swiss-Prot (Additional file 2: Table S9). No sequences in EMBL-EBI were identified with a BTK motif and TIR domain and only two sequences containing both a TIR_2 and BTK motif were found in EMBL-EBI. These predicted proteins were found in *Phytophthora ramorum* (oomycete) and *Guillardia theta* (crytpomonad algae) (UniProt IDs in Additional file 2: Table S10). NG2 is represented in Fig. 2b.

Novel Gene 3 (NG3) was identified in only three species, *A. pallida, C. polypus* and *N. vectensi*s. NG3 includes the Pfam domains protein tyrosine kinase or protein kinase (Pkinase_Tyr or Pkinase; always overlapping), a death domain and a TIR (or TIR_2) domain. Additional domains are seen in this gene when represented by SMART; these included central Roc/COR domains and a SH3 domain (SRC Homology 3 domain). Only a single *Nematostella* predicted protein was found to match this transcript in the TrEMBL database (Additional file 2: Table S9). BLASTx analyses against the Swiss-Prot database (Additional file 2: Table S9) identified two slime mould predicted proteins with similarity; however, neither contained either the DEATH or TIR domains, and were therefore disregarded. EMBL-EBI database search returned no TIR domain architectures that were similar to NG3. A few sequences were found in the EMBL-EBI database with TIR_2 domain combinations similar to NG3. The most similar domain combination found was with a ‘neuralized’ domain in place of the ‘Pkinase’ and ‘SH3’ domains in *Strigamia maritima* (centipede) and *Crassostrea gigas* (pacific oyster). The ‘Pkinase’ and ‘SH3’ domains are also missing in a less similar domain architecture found in *Strongylocentrotus purpuratus* (purple sea urchin) (Additional file 2: Table S10). NG3 is represented in Fig. 2c.

### Evolutionary and Phylogenetic analyses

#### Non-synonymous vs synonymous substitution rates

Non-synonymous vs synonymous substitution rates (d_N_/d_S_) were calculated using PAML for all *TLR*, *MyD88*, *NF-κB* and TMD containing *NLR*s genes, as well as for Novel Genes 1, 2 and 3. Table 3 shows a summary of the results and all PAML results for the Yang and Nielsen [33] method can be viewed in Additional file 4. Conserved and novel genes where all found to have patterns of synonymous and non-synonymous mutations consistent with the action of purifying selection (i.e., dN/dS ratio < 1) across all genes.

**Table 3:** Non-synonymous vs synonymous substitution rates

Gene	d_N_ (±SE)	d_S_ (±SE)	d_N_/d_S_
*TLR*	0.3610 (±0.0162)	1.5845 (± 0.2657)	0.2278
*MyD88*	0.3140 (± 0.0298)	2.4488 (± 1.1913)	0.1283
*NF-κB*	0.2938 (± 0.0299)	2.4713 (± 2.9559)	0.1189
TMD containing NLRs	0.5990 (± 0.0244)	1.8720 (± 0.6182)	0.3200
Novel Gene 1	0.1757 (± 0.0083)	1.4509 (± 0.1338)	0.1211
Novel Gene 2	0.2480 (± 0.0145)	2.2503 (± 0.4263)	0.1102
Novel Gene 3	0.3757 (± 0.0144)	3.9824 (± 8.6207)	0.0943

All values show as averages for each gene, calculated using the Yang and Nielsen [33] method. Average d_N_/d_S_ ratio does not take into account standard error

### NLR family phylogenetic analysis

**Fig. 3:**
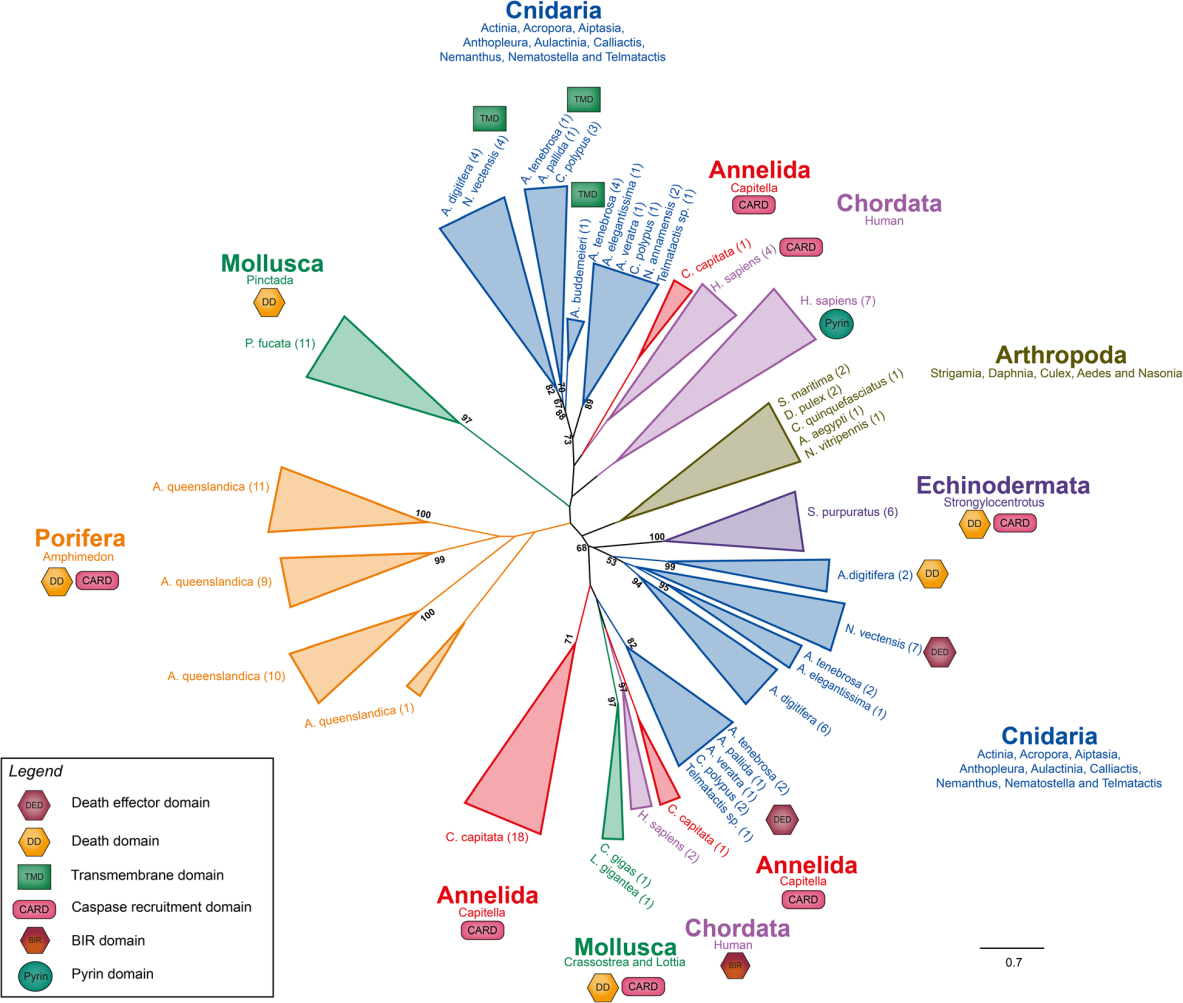
Phylogenetic analysis of metazoan NLR genes. Maximum likelihood analysis was performed from an alignment of the conserved NACHT domains (aa) from multiple metazoan species. Only bootstrap values more than 50 were retained for clades of interest. Domains also present in the full NLR sequence are depicted next to each clade. Clades are colour-coded by phylum and labelled with the phylum name, with the genera present in that clade underneath. Anthozoan NLR clades are highlighted on the tree, with the number of sequences for each species in the clade shown in brackets. Clades with no domain symbol have the canonical NLR architecture (one NACHT domain and multiple LRR)

Maximum likelihood analysis was used to infer phylogenetic relationships of NLRs between actiniarian species in this study with a selection of additional metazoan NLRs. Figure 3 shows a radial maximum likelihood tree, generated using a LG + G protein model. Highlighted on the tree are two clades, which correspond to NLRs with certain classes of N-terminal domains. All known metazoan TMD containing NLRs cluster into one clade, which only consists of anthozoan species (Actiniaria and Scleractinia (*A. digitifera*)). Most of the other anthozoan NLRs all cluster into another clade. Interestingly the DED sequences from anemone species from this study cluster more closely with the Chordata, Annelida and Mollusca BIR, CARD and DD clade, than with other anthozoan species (i.e., *N. vectensis* and *A. digitifera*), although this is lowly supported*. N. vectensis* NLRs consistently cluster outside of other actiniarian species with high bootstrap support.

### CniFL family phylogenetic analysis

**Fig. 4:**
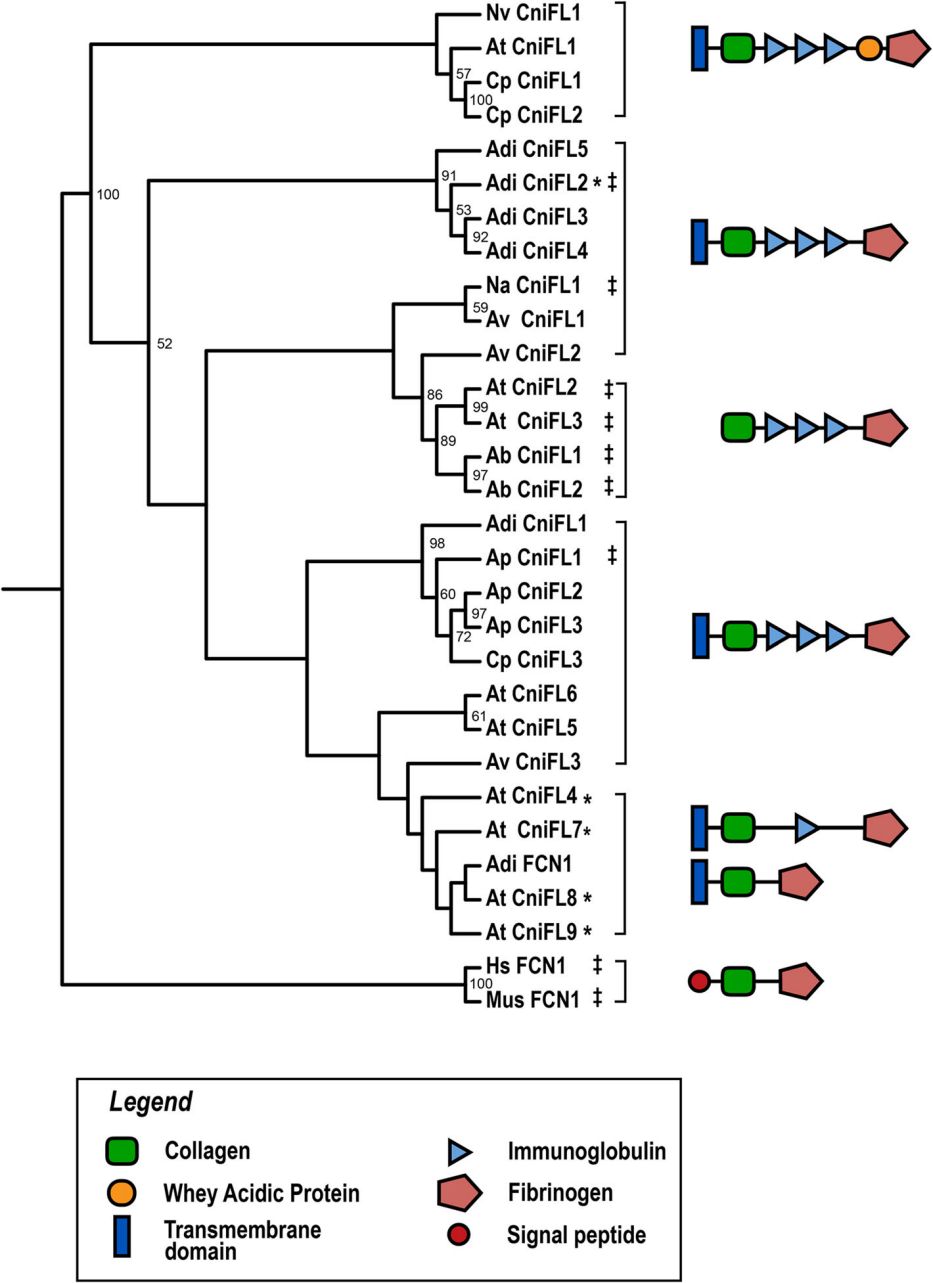
Phylogenetic analysis of anthozoan CniFL genes. Maximum likelihood analysis was performed from an alignment of the ORF (aa), which was trimmed to remove poorly aligned sequence, resulting in a 132 aa alignment. Bootstrap values below 50 are removed. In most instances, one TMD domain and three Ig domains were identified; asterisks denote sequences with only one Ig domain and ‡ denote sequences with no TMD. CniFL sequences are numbered within each species; however, not all CniFLs identified in this study are shown in the tree, as duplicate trimmed sequences were removed from the alignment. Style of domains shown is adapted from [27] in order to facilitate comparison between figures. Species named as follows: Adi, *A. digitifera*; At, *A. tenebrosa*; Ap, *A. pallida*; Ab, *A. buddemeieri*; Av, *A. veratra*; Cp, *C. polypus*; Hs, *Homo sapiens*; Mus, *Mus musculus*; Na, *N. annamensis*; Nv, *N. vectensis*

A total of 42 protein sequences from all species except *A. elegantissima* and *Telmatactis* sp, were identified with a complete ORF. Removing duplicate sequences resulted in a final alignment of 30 sequences, which was trimmed to remove poorly aligned regions to a final 132 aa alignment. The phylogeny represented in Fig. 4 was generated using the JTT + G protein model of evolution and the alignment used can be found in Additional file 5. Most CniFL genes were identified with an additional TMD which is represented in the domain architecture illustrations in Fig. 4. One clade also contains CniFLs with an additional domain WAP (whey acidic protein) found in *N. vectensis, A. tenebrosa* and *C. polypus*. Mapping back the *N. vectensis* CniFL transcript to the genome (v1.0) identified the *N. vectensis CniFL* gene at scaffold_24:467371-486150 (gene model: e_gw.24.49.1). Multiple Ig domains were annotated by Pfam in the genome; however, they are annotated in a region that has been annotated as intronic.

## Discussion

To better understand the evolution of the innate immune system we have performed a comprehensive and comparative investigation of the actiniarian innate immune gene repertoire, including *TLR, NLR,IL-1R*-like, *MyD88, NF-κB, CniFL, SRCR, MASP,* C-type lectin domains, complement control proteins and multiple novel innate immune genes. By generating multiple high-quality transcriptome assemblies we were able to undertake a detailed study of innate immune genes in actiniarians. Here we note that in some cases specific gene families appear to have undergone lineage-specific expansions and potential neofunctionalisation (e.g. *NLR*), while other genes such as *TLR*s have fewer copies than observed in other eumetazoan phyla. Although these observations need further functional validation, they provide an insight into the core eumetazoan immune gene set and show that the majority of actiniarian innate immune genes show evidence of purifying selection.

### Candidate and novel gene counts and architectures

The identification of candidate and novel genes in this study is mostly consistent with previously reported information on gene copy number in cnidarians. For example [11], reported similar counts for some TIR domain containing proteins identified in nine anthozoan species. Their study included three of the species interrogated in our study: *A. pallida, A. elegantissima* and *N. vectensis* and confirmed single copies of *TLR* and *MyD88*. For *IL-1R-*like, of which there are multiple copies, fewer complete genes were identified in our study for the three species, whilst numbers of all TIR domain containing proteins were higher. The study [11] also identified an expansion of TIR only proteins restricted to coral species. Here we present evidence for an anthozoan-specific expansion of TIR only proteins, as we identify similar counts of TIR only proteins in all anemone species (Additional file 2: Table S8).

In this study, the TIR domain, not TIR_2, was always found in proteins with evolutionarily conserved architectures (i.e., TLR, MyD88, IL-1R-like). Notably, in most instances TIR_2, a common bacterial TIR domain [34] also found in some marine invertebrates [35–37] was found in novel genes identified here. This highlights that horizontal gene transfer (HGT) may have played a role in the introduction of the TIR_2 domain to the genome of actiniarian species, which then underwent domain shuffling to form the observed novel genes. Whilst the possibility of contamination cannot conclusively be ruled out, in most instances no similar domain architectures to the three novel genes were identified in any other species. This study identifies two previously uncharacterised genes (NG1 and NG2) and extends on the previously reported novel genes for Actiniarians [11], including NG3 and other novel genes shown in Fig. 2, which have been identified by [11]. Interestingly, NG3 identified here was found with additional domains (SH3, Roc and COR domains) not identified by [11]. These novel domain architectures found in lineage-specific actiniarian species are likely the result of domain shuffling.

### Expanded repertoire and neofunctionalisation of NLRs

The diversity in domain architectures observed in the NLR family across metazoans suggests potential evidence for neofunctionalisation in this gene family. The expansion and diversification of actiniarian NLRs, such as those with transmembrane domains, observed in our study is significant as it indicates that some of these duplicated copies have likely undergone independent neofunctionalisation within Anthozoa. This is supported by phylogenetic analysis of NLRs across Metazoa (Fig. 3) where all 15 TMD containing NLRs clustered into one clade, but other actiniarian NLRs clustered in separate clades. The presence of multiple transmembrane domains in NLRs identified in our study indicates that they may be membrane-bound. This appears to be an anthozoan specific innovation as all other metazoan NLRs are exclusively localised to the cytoplasm and do not have these domains [38]. Interestingly, only two other anthozoan species (*N. vectensis* and *A. digitifera)* have so far been reported with TMD containing NLRs [7], but our results indicate a wide distribution of TMD containing NLRs across multiple actiniarian species. This highlights that novel innovations and neofunctionalisation have occurred in innate immune gene families of Actiniaria. *Amphimedon queenslandica* (sponge) NLRs may also contain TMDs, however, no anthozoan TMD containing NLRs cluster with these sequences and do not currently support this hypothesis [7]. Phylogenetic analysis of the metazoan NLRs resulted in a similar tree to the one obtained by [7] in their study on sponge NLRs, providing further evidence that the TMD containing NLR clade are correctly clustered into a single clade, indicating this novel structure has likely evolved from a single origin within Anthozoa. Potential functional implications of the neofunctionalisation of NLRs include allowing cooperation between other pattern recognition receptors or a modified or novel response to an immune signal.

### Conservation of innate immune pathways and the interaction of novel genes

A functioning innate immune system may depend on the conservation of signalling pathways, more so than the conservation of particular immune genes. For example, in *Hydra* the TLR pathway is conserved through a scaffold of TIR-only and LRR-only proteins despite the lack of a full length *TLR* gene [18–20]. In our study, however, we found canonical *TLR* genes in most species (Table 1), with the exception of *A. pallida* and *Telmatactis* sp. In fact, no canonical *TLR* gene was identified in the *Aiptasia* genome [27]. Whether *A. pallida* and *Telmatactis* sp. also use scaffold TIR-only and LRR-only proteins remains unexamined, however, the expansion of TIR-only and TIR-domain-containing proteins in all species in this study may contribute to the building of scaffold-like immune pathways*.* Interestingly, the presence of 4 full length canonical *TLR* genes in coral (i.e., *A. digitifera*) [25] suggests that the canonical *TLR* was present in the common ancestor of anthozoans.

This principle of pathway conservation is demonstrated by the evolution of the interleukin-1 receptor gene family. The Ig-TIR domain combination (found in interleukin receptors such as IL-1R) is hypothesised to have evolved independently in both cnidarians and vertebrates [11, 12]. No IL-1R-like identified here were annotated as bona fide IL-1R protein by Swiss-Prot, rather they were all annotated as either TIR-domain-containing proteins or Ig containing genes. Further domain analysis demonstrated that these genes do indeed possess the canonical IL-1R-like architecture, with a C-terminal TIR, transmembrane domain and multiple Ig domains (Fig. 1). Previous studies [5, 11] have reported the independent evolution of the Ig-TIR domain combination and thus concluded that cnidarian *IL-1R* (or rather *IL-1R*-like,) are not orthologs of the vertebrate *IL-1R* family. In particular, one study [12] argued that either the LRR-TIR (as seen in TLRs) or the Ig-TIR domain combination must have evolved independently in more than one metazoan lineage. Further phylogenetic analyses may clarify whether these gene families or domain combinations have evolved independently in multiple metazoan lineages.

Expansions in genes encoding TIR-domain-containing proteins, including both novel and ‘conserved’ (i.e., IL-1R-like) genes, may compensate for the lack of more than one *TLR* (or no *TLR*, in the case of *Aiptasia*, *Hydra* and *Telmatactis* sp. [19, 27]). The TLR and IL-1R-like pathways both interact with MyD88 via their TIR domain during signal transduction, and therefore, may also interact with novel TIR-domain-containing proteins in a similar way. It is plausible to hypothesise that Novel Gene 3 (Fig. 2c) may interact in these pathways via its C-terminal death and TIR domains, which together form the canonical MyD88 domain architecture. Other novel TIR-domain-containing proteins (Fig. 2) may also interact in known or new pathways via the interactions of the TIR domain. Functional validation is required to determine the extent to which these novel genes contribute to cellular processes underpinning the innate immune response.

### Distribution of CniFL: a putative complement pathway receptor

The initiation of known immune pathways though novel or lineage-specific immune genes is demonstrated by the discovery of the novel putative immune receptor CniFL. First reported in the recently sequenced *Aiptasia* genome [27], CniFL is hypothesised to function through the lectin-complement pathway. Here we identify a copy of the *CniFL* gene (Table 2) in all species (except *A. elegantissima,* however, this transcriptome was derived from a single tissue source (acrorhagi)), as well as multiple components of the complement pathway (Table 2). We also identify three species (*N. vectensis, A. tenebrosa* and *C. polypus*) with CniFLs that contain an additional WAP domain and also identify a transmembrane domain CniFL in most species (Fig. 4). Interestingly, the single *CniFL* gene copy in *N. vectensis* was not identified previously [27] due to an incorrectly annotated ab initio gene model prediction in the *Nematostella* genome (v1.0). In addition, *CniFL* appears to have extensive copy number variation within hexacorallians (1 in *N. vectensis* to 7 in *A. tenebrosa*), but whether this variation in copy number is adaptive remains unexplored. The *CniFL* gene was hypothesised to have a role in symbiont uptake [27], which was partly due to its absence in the non-symbiotic *N. vectensis*. However, we observe that the *CniFL* gene is present in both symbiotic and non-symbiotic (*A. tenebrosa, A. buddemeieri, C. polypus, N. vectensis*) species investigated in our study (see Additional file 1: Table S2) with highest copy number reported in a non-symbiotic species (*A. tenebrosa*), therefore we recommend further functional validation to support this hypothesis.

## Conclusion

In conclusion, actiniarians have a diverse repertoire of innate immune genes. These include novel domain architectures and potentially novel innovations, such as membrane-bound NLRs and novel putative complement pathway PRRs (i.e., CniFL). Further novelties may have been introduced to the actiniarian immune gene set through HGT followed by domain shuffling. Together, the conservation, expansion and diversification of different gene families and genes encoding TIR-domain-containing proteins have shaped the evolution of the actiniarian innate immune system. The conservation of specific protein domain architectures, immune genes and pathways provides an insight into the core gene set required for a functioning eumetazoan innate immune system. Further functional validation of these genes may elucidate conserved and novel biological functions, including how these genes may contribute to lineage-specific processes and morphological novelties.

## Methods

To perform a comparative analysis of the innate immune gene repertoire, across multiple actiniarian taxa, we interrogated a suite of genomic resources, both publically available and newly generated in this study. Specifically, genomic datasets used in this study consisted of newly sequenced transcriptomes for *Actinia tenebrosa* (total *n* = 5; red colourmorph *n* = 2, brown *n* = 1, green *n* = 1, blue *n* = 1)*, Anthopleura buddemeieri* (*n* = 1), *Aulactinia veratra* (*n* = 2), *Calliactis polypus* (*n* = 2), *Nemanthus annamensis* (*n* = 1) and *Telmatactis* sp*.* (*n* = 1), and as well as publically available transcriptomes for *Anthopleura elegantissima* (*n* = 1; SRX754678), *Aiptasia pallida* (*n* = 1; SRX231866, run SRR696721) and *Nematostella vectensis* (*n* = 1; SRX315372). These species were selected based on their phylogenetic distribution across most superfamilies within Actiniaria, availability of animals and general lack of sequence information for these species. Multiple transcriptomes were generated for *A. tenebrosa* and *A. veratra* which represent colourmorphs (red, brown, green and blue for *A. tenebrosa*) and aerial exposure treatments (one control and one treatment each for *A. tenebrosa* (red only) and *A. veratra*). All raw sequence reads were submitted to NCBI Sequence Read Archive under BioProject accession PRJNA313244 (see Additional file 1: Table S1 for all accession numbers).

### Animal acquisition

Individual specimens of *A. tenebrosa, A. buddemeieri* and *A. veratra* were collected from the intertidal zone at Point Cartwright, (QLD, Australia) in 2014. *C. polypus* individuals were collected in 2014 from pumice washed onto rock pools at Snapper Rocks (QLD, Australia). *Telmatactis* sp. was bought from Cairns Marine (QLD, Australia) in 2014. *N. annamensis* was bought from Great Barrier Reef Marine Pty Ltd in 2015. All animals were housed in holding tanks, in the marine laboratory at QUT, until experimental use. Tanks were maintained at between 33 and 37 ppt salinity and 20–28 °C.

### Sequencing, assembly and annotation

Total RNA was extracted from whole organisms by first homogenizing individuals in liquid nitrogen, followed by a TRIzol/chloroform RNA extraction protocol (TRIzol®, Life Technologies). Extracted RNA was assessed for quality and integrity on a Bioanalyzer 2100 (Agilent) using an RNA nano chip. Sequencing libraries were prepared using the Illumina TruSeq® Stranded mRNA Library Preparation Kit and sequenced on an Illumina NextSeq 500. 150 bp paired-end chemistry was used for all sample libraries, except *N. annamensis* and *A. tenebrosa* (4; blue) which were prepared at a different time using 75 bp paired-end chemistry. All transcriptomes were assembled using the Trinity *de novo* assembler [39]. Trinity assembler was used for *A. tenebrosa* (1, 2, 3 and 4), *A. pallida, A. buddemeieri, A. elegantissima, A. veratra, C. polypus* (1, 2)*, N. annamensis, N. vectensis* and *Telmatactis* sp.. Quality trimming was performed on all datasets using Trimmomatic [40] with default settings to retain only high quality reads and to remove non-biological sequence.

Redundant and chimeric sequences were removed from all assemblies using CD-Hit v.4.6.1 [41, 42], by clustering sequences with greater than 95 % similarity within each assembly. In addition, CEGMA (Core Eukaryotic Genes Mapping Approach) v.2.5 [43] was used to assess the completeness of each assembly, by determining the percentage of full-length sequences in each transcriptome corresponding to 248 highly conserved eukaryotic proteins.

Functional annotation of individual transcriptomes was undertaken using Trinotate [44], for both nucleotide and predicted peptide sequences. Nucleotide sequences for each contig were annotated using BLASTx searches against the Swiss-Prot [45] and TrEMBL (Uniref90) [46] databases using BLAST+ v.2.2.31 [47, 48] software (E value 1 × 10^−5^). Batch extraction and translation of the longest open reading frames (ORFs) for each contig were performed using TransDecoder v.2.0.1 [49, 50]. From the predicted peptide sequences, signal peptides and protein families were detected in each ORF, using Signal Peptide v.4.1 [51] and Pfam v.3.1b1 [32], respectively. Longest ORFs were annotated using BLASTp searches against the Swiss-Prot [45] and TrEMBL [46] databases using BLAST+ v.2.2.31 [47, 48] software (E value 1 × 10^−5^).

### Gene ontology and RSEM analysis

GO terms were assigned to contigs that received significant BLASTx hits (E value 1 × 10^−5^). WEGO [52] and CateGOrizer [53] were used to examine the distribution of top GO terms and the distribution of ‘immune class’ GO terms in each assembly, respectively. Estimates of transcript abundance for all newly generated transcriptomes were calculated using the Trinity [50] pipeline. Raw reads were aligned to assembled contigs using Bowtie 2 [54], followed by RNAseq by Expectation-Maximization (RSEM) [55] estimation of expression values as Fragments per Kilobase of transcript per Million mapped reads (FPKM).

### Identification of candidate genes

The list of candidate genes interrogated in this project was limited to five innate immune gene families which included Toll-like receptor (*TLR*), Nucleotide-binding and Leucine-rich Repeat containing gene (*NLR*), Interleukin-1 receptor-like genes (*IL-1R-*like), Myeloid Differentiation primary response gene 88 (*MyD88*) and Nuclear Factor kappa-light-chain-enhancer of activated B cells (*NF-κB*). Transcripts containing full length ORFs of these candidate genes were identified using Pfam, BLASTp and BLASTx searches. Specifically, the search method for each candidate gene was as follows. *TLR* was identified by searching for all contigs with a TIR (PF01582) (or TIR_2, PF13676) domain, along with at least one leucine-rich repeat (LRR, CL0022). *MyD88* was identified by searching for a TIR (or TIR_2) domain, along with a death domain (DD). *IL-1R-*like was identified by searching for a TIR (or TIR_2) domain, along with least one immunoglobulin (Ig, CL0011) domain. *NF-κB* was identified by searching for a Rel homology domain (RHD, PF00554), along with at least one ankyrin repeat (PF00023). The presence of other domains with a RHD may be indicative of different classes of *NF-κB*, however, in these actiniarian species no other domains were found in the same contig with a RHD. Hence, all identified *NF-κB* belong to Class 1. A Rel homology domain without ankyrin repeats, as seen in *N. vectensis* [56] were not considered as full length ORFs. *NLR* was identified by searching for a NACHT (domain found in NAIP, C2TA, HET-E and TP1) domain, along with at least one LRR. *NLR* genes may also include a variable N-terminal domain, which can be broadly classified in the ‘death-fold superfamily’ which includes the domains caspase activation and recruitment domain (CARD, PF00619), pyrin domain (PYD, PF02758), death domain (DD, PF00531) and death effector domain (DED, PF01335). Therefore, contigs also containing a domain from this superfamily were considered potential candidate *NLR*s. In addition, the translated ORF for each candidate gene was analysed by TMHMM v.2 [57], to identify transmembrane domains.

Finally, contigs were only retained as complete candidate genes if they consisted of a full length ORF which contained a start and stop codon, and received a BLASTp hit (from either the TrEMBL or Swiss-Prot databases) against other species using the open reading frame batch extracted by TransDecoder [49].

### Presence/absence of other innate immune genes

Non-conservative counts of other innate immune genes in this study were identified from gene counts provided by Trinotate. CniFL was identified by searching for all genes containing the Pfam domains Collagen (PF01391), Ig (CL0011) and Fibrinogen (PF00147). MASP was identified from BLAST hits from TrEMBL with *N. vectensis* MASP and presence of the correct Pfam domains (CUB, EGF-like calcium binding, CUB2, Sushi 1, Sushi 2 and Peptidase S1). SRCR were identified by searching for the number of genes with Pfam annotations for the SRCR domain (PF00530). C-type lectin domains were identified by searching for the number of genes with Pfam annotations for lectin_C domains (PF00059). Complement control proteins (C3, Factor B, C6 and Factor I) were identified by searching for BLAST hits from either the TrEMBL or Swiss-Prot databases.

### Identification of novel immune genes

Potential novel genes were identified by searching for novel domain architectures that included the TIR (PF01582) and/or TIR_2 (PF13676) domains. This domain is well documented in having a role in signal transduction and protein-protein interactions in immune pathways, and therefore, provides a robust target for finding putative novel genes that interact in innate immune pathways [58]. All species were also interrogated for novel actiniarian genes previously identified [11, 27]. Initially, all TIR or TIR_2 domain containing contigs, for each species, were identified in the Trinotate annotation report, and all contigs without a BLASTx or BLASTp annotation were further investigated. In addition, contigs that received BLASTx or BLASTp hits only against *N. vectensis* predicted proteins or genes were also interrogated in detail. The EMBL-EBI [32] Pfam domain architecture analysis (http://pfam.xfam.org/ Accessed 28^th^ March 2016) was used to investigate known architectures with either the TIR or TIR_2 domains, and thereby identify which species, if any, have genes with novel or infrequently used architectures. Novel architectures were identified if no or few hits were returned from either BLAST or EMBL-EBI, which may indicate potential actiniarian lineage-specific (orphan) genes, horizontally transferred genes or taxonomically-restricted genes.

### Candidate and novel gene validation

To validate the transcriptome assemblies, as well as the ORF of candidate transcripts, PCR validation was performed for the five candidate genes, as well as three novel genes and *CniFL*. All primers were designed using Primer-BLAST [59] using the settings of [60], to amplify the ORF of each respective gene for four species: *A. tenebrosa, A. buddemeieri*, *A. veratra* and *C. polypus* (except *CniFL*, for which genes were validated in *A. tenebrosa* and *A. buddemeieri* only)*.* For large ORFs, multiple primer sets were designed to ‘walk’ the length of the ORF, with at least 100 bp overlap between products. A detailed list of primer sequences is presented in Additional file 3: Table S11. PCRs were performed using RNA extracted using the above described protocol, followed by cDNA synthesis using the SensiFAST™ cDNA synthesis kit (Bioline). PCR products were then amplified using MyFi™ DNA polymerase mix (Bioline) (PCR protocols and thermocycler conditions can be found in Additional file 3: Tables S12–S13). Purification was performed using the ISOLATE II PCR and Gel kit (Bioline), and sequenced using BigDye® Terminator v3.1 (ThermoFisher). Sequences were run on a Genetic Analyser 3500 from Applied Biosystems (ThermoFisher). Sequence chromatograms were visualised in Geneious version R8.1.4 [61] and aligned to the transcript from which the primers were designed, as per [62]. To confirm the ORF for the candidate gene, sequence similarity was compared.

### Evolutionary and phylogenetic analyses

#### PAML

To investigate whether genes were under purifying, neutral or positive selection and thereby provide insights into the selective pressures on the actiniarian immune system, PAML analysis was performed. Non-synonymous vs synonymous substitution rates (d_N_/d_S_) of all *TLR*, *MyD88*, *NF-κB* and the transmembrane domain containing *NLR*s, as well as Novel Genes 1, 2 and 3, were analysed using the method of Yang and Nielsen [33]. First, the nucleotide sequence for the complete ORF of all genes were extracted from the TransDecoder output and imported into Geneious [61] version R8.1.4. All stop codons were manually removed and then sequences were imported into MEGA version 7.0.14 [63] for alignment with MUSCLE [64] (codon), using default settings and gaps removed. Alignments were converted into PHYLIP format in Geneious [61] and then imported into pamlX [65, 66] version 1.3.1, where d_n_/d_s_ ratios were calculated using YN00 [33]. All values for d_N_ and d_S_ were averaged for each gene to provide an average d_N_/d_S._

#### RAxML

In order to understand how actiniarian NLRs have evolved in the context of the metazoan NLR family, the phylogenetic relationship of NLRs in this study and other metazoan NLRs were resolved using maximum likelihood analysis in RAxML. Of particular interest was the question of how the novel TMD containing NLRs have evolved (i.e., from a single origin or multiple independent origins). Initially, the translated ORFs for 35 complete and near-complete NLRs (near-complete only used for species where no complete NLR found) of the candidate NLR transcripts were imported into Geneious [61] version R8.1.4 and aligned along with NACHT domains from species across Metazoa (Additional file 6) which were obtained from [7]. Alignments were performed using MUSCLE [64] with default settings (duplicates removed). Sequences were trimmed to retain only the NACHT domain, as this represents the most evolutionary conserved domain of NLRs, across metazoan species and is useful for inferring phylogenetic relationships [7]. Alignments were imported into MEGA version 7 [63] to determine the best model of protein evolution. Maximum likelihood analysis was then performed in RAxML version 8.1.15 [67], using rapid bootstrapping (1000 replicates) with a random seed and no outgroup.

To understand how *CniFL* genes are disturbed across Anthozoa species, maximum likelihood analysis of CniFL proteins was performed. All but two species had at least one gene copy for which a complete ORF was identified; *Telmatactis sp*. had only partial sequences and no *CniFL* was identified in *A. elegantissima*. An initial alignment of the amino acid sequences of the complete ORFs was performed using MUSCLE [64]. Alignments were trimmed using Gblocks [68] to remove poorly aligned sections, allowing for gap positions within final blocks and not allowing many contiguous non-conserved positions. Protein model testing in MEGA [63] and maximum likelihood analysis in RAxML [67] was performed the same as above for NLR. The outgroups (Hs FCN1 and Mus FCN1) were set post-analysis in Geneious [61]. SMART analysis [69, 70] was used to identify the number of domains in each CniFL shown in the tree.

## Additional files

Additional file 1: Transcriptome sequencing, assembly and annotation. **Tables S1**–**S5** and **Figure S1**. Data accession numbers, assembly statistics and annotation results, including GO term analyses using CateGOrizer and WEGO. (DOCX 3213 kb)
Additional file 2: Identification and characterisation of candidate and novel innate immune genes. **Table S6**–**S10**. Counts of number of complete and partial candidate and novel innate immune genes, novel gene BLAST hits, domain architectures and database queries. (DOCX 46 kb)
Additional file 3: PCR validation of candidate and novel genes. **Tables S11**–**S14**. Primer lists, PCR protocols and GenBank accession numbers. (DOCX 32 kb)
Additional file 4: PAML results using the Yang and Nielsen [33] method. PAML results for complete sequences only of TLR, MyD88, NF-κB, TMD-containing NLRs, Novel Gene 1, Novel Gene 2 and Novel Gene 3. (XLSX 46 kb)
Additional file 5: Alignment used for *CniFL* genes phylogenetic analysis. (FASTA 4 kb)
Additional file 6: Alignment of NACHT domains from metazoan species. (FASTA 36 kb)
