# Supplementary material for: Insights into the innate immunome of actiniarians using a comparative genomic approach
Source: BMC Genomics. 2016 Nov 2;17:850. doi: 10.1186/s12864-016-3204-2 (PMC5094078; doi:10.1186/s12864-016-3204-2)
Supplement: Additional file 1: — Transcriptome sequencing, assembly and annotation. Tables S1–S5 and Figure S1. Data accession numbers, assembly statistics and annotation results, including GO term analyses using CateGOrizer and WEGO. (DOCX 3213 kb) [file 12864_2016_3204_MOESM1_ESM.docx]

# Additional file 1 | Transcriptome sequencing, assembly and annotation

**Insights into the innate immunome of actiniarians using a comparative genomic approach**

Chloe A. van der Burg^1,2^, Peter J. Prentis^3,4^ Joachim M. Surm^1,2^ and Ana Pavasovic^1,2^

^1^School of Biomedical Sciences, Faculty of Health, Queensland University of Technology, GPO Box 2434, Brisbane, Qld 4000

^2^Institute of Health and Biomedical Innovation, Queensland University of Technology, GPO Box 2434, Brisbane, Qld 4000

^3^School of Earth, Environmental and Biological Sciences, Science and Engineering Faculty, Queensland University of Technology, GPO Box 2434, Brisbane, Qld 4000

^4^Institute of Future Environments, Queensland University of Technology, GPO Box 2434, Brisbane, Qld 4000

Corresponding author: Chloe van der Burg.

Email: chloe.vanderburg@hdr.qut.edu.au

# Supplementary results

## Sequencing, assembly and annotation

**Table S1. Sequence read archive and BioSample accession numbers.** All samples submitted under one BioProject (accession number PRJNA313244.). All SRA submissions study accession number is SRP070917. Note that for samples where treatments were applied the reads were assembled together as one transcriptome (i.e., *A. tenebrosa* (red) and *A. veratra*).

| **Transcriptome** | **Other details** | **BioSample** | **SRA accession** |
| --- | --- | --- | --- |
| *A tenebrosa* (1) | Control. Red colour-morph | SAMN04520502 | SRR3216075 |
|  | Treatment. Red colour-morph | SAMN04520715 | SRR3193648 |
| *A. tenebrosa* (2) | Brown colour-morph | SAMN04534887 | SRR3207346 |
| *A. tenebrosa* (3) | Green colour-morph | SAMN04534878 | SRR3206038 |
| *A. tenebrosa* (4) | Blue colour-morph | SAMN04534877 | SRR3207346 |
| *A. buddemeieri* | - | SAMN04521008 | SRR3205971 |
| *A. veratra* | Control | SAMN04523369 | SRR3205707 |
|  | Treatment | SAMN04523371 | SRR3205708 |
| *C. polypus* (1) | 2015 sample | SAMN04529561 | SRR3205709 |
| *C. polypus* (2) | 2014 sample | SAMN04529559 | SRR3205762 |
| *N. annamensis* | - | SAMN04534889 | SRR3228732 |
| *Telmatactis* sp. | CAVDB-2016 | SAMN04534888 | SRR3225580 |

**Table S2. Assembly statistics**. Table shows assembly data for all 13 transcriptomes used in this study. Data in brackets represents values after CD-Hit was performed. Details of transcriptomes included whether the anemone species is normally symbiotic with zooxanthellae, non-symbiotic, or if an aposymbiotic strain was used for RNAseq. Table continues over next two pages.

|  | ***Actinia tenebrosa* (1)** | ***Actinia tenebrosa* (2)** | ***Actinia tenebrosa* (3)** | ***Actinia tenebrosa* (4)** |
| --- | --- | --- | --- | --- |
| Source | whole organism | whole organism | whole organism | whole organism |
| Details | red (n=2)  Non-symbiotic | brown (n=1)  Non-symbiotic | green (n=1)  Non-symbiotic | blue (n=1)  Non-symbiotic |
| Treatment | 1 = Control, 2 = 3 hours air exposure  (reads combined into one transcriptome) | Control | Control | Control |
| Number of reads | 152,136,760 | 201,995,450 | 179,309,262 | 175,687,690 |
| Total assembled base pairs | 128,035,388 (88,116,072) | 121,697,534 (98,585,782) | 105,742,119 (86,177,706) | 113,760,033 (83,348,231) |
| Number of transcripts | 166,589 (111,882) | 147,092 (122,362) | 126,534 (105,145) | 116,930 (87,137) |
| N10 | 4,670 bp (4,799 bp) | 5,421 bp (5,215 bp) | 5,218 bp (5,054 bp) | 5,192 bp (5,108 bp) |
| N30 | 2,451 bp (2,558 bp) | 2,879 bp (2,756 bp) | 2,816 bp (2,702 bp) | 2,889 bp (2,846 bp) |
| N50 | 1,390 bp (1,478 bp) | 1,680 bp (1,600 bp) | 1,682 bp (1609 bp) | 1,804 bp (1,770 bp) |
| Maximum length | 30,441 bp | 32,195 bp | 30457 bp | 31384bp |
| Average contig length | 769.57 bp (787.58 bp) | 827.36 bp (805.69 bp) | 835.7 bp (819.6 bp) | 972.89 bp (956.52 bp) |
| **CEGMA (before CD-Hit)** |  |  |  |  |
| Full length (%) | 92.4 | 95.6 | 96.8 | 97.98 |
| Full length and partial (%) | 96.4 | 98.4 | 98.4 | 98.79 |
| **CEGMA (after CD-Hit)** |  |  |  |  |
| Full length (%) | 92.3 | 95.6 | 96.8 | 97.98 |
| Full length and partial (%) | 96.4 | 98.4 | 98.4 | 98.79 |

|  | ***Aiptasia pallida*** | ***Anthopleura buddemeieri*** | ***Anthopleura elegantissima*** | ***Aulactinia veratra*** |
| --- | --- | --- | --- | --- |
| Source | whole organism | whole organism | acrorhagi | whole organism |
| Details | SRX231866, run SRR696721  Aposymbiotic | n=1  Non-symbiotic | SRX754678  Symbiotic | n=2  Symbiotic |
| Treatment | Control | Control | Control | 1 = Control, 2 = 3 hours air exposure  (reads combined into one transcriptome) |
| Number of reads | 220,632,160 | 51,620,970 | 189,025,968 | 69,513,111 |
| Total assembled base pairs | 117,124,411 (93,034,971) | 141,560,960 (95,119,754) | (157,977,349) | 129,509,749 (88,375,948) |
| Number of transcripts | 124,850 (109,430) | 212,774 (145,473) | 199,194 (186,219) | 174,203 (118,019) |
| N10 | 5,961 bp (5,792 bp) | 3,511 bp (3,633 bp) | (4434 bp) | 4,449 bp (4,663 bp) |
| N30 | 3,331 bp (3,190 bp) | 1,846 bp (1,873 bp) | (2318 bp) | 2,331 bp (2,430 bp) |
| N50 | 2,017 bp (1,859 bp) | 1,034 bp (1,025 bp) | 1,583 bp (1,487 bp) | 1,333 bp (1,407 bp) |
| Maximum length | 36,549 bp | 29,353 bp | 65,222 bp | 30,277 bp |
| Average contig length | 938.12(850.18 bp) | 665.31 bp (653.87 bp) | (848.34 bp) | 743.44 bp (748.83 bp) |
| **CEGMA (before CD-Hit)** |  |  |  |  |
| Full length (%) | N/A | 88.7 | N/A | 95.6 |
| Full length and partial (%) | N/A | 97.2 | N/A | 98.4 |
| **CEGMA (after CD-Hit)** |  |  |  |  |
| Full length (%) | 95.6 | 88.7 | 98.8 | 95.6 |
| Full length and partial (%) | 97.2 | 96.8 | 99.1 | 98.4 |

|  | ***Calliactis polypus*** **(1)** | ***Calliactis polypus* (2)** | ***Nemanthus annamensis*** | ***Nematostella vectensis*** | ***Telmatactis* sp.** |
| --- | --- | --- | --- | --- | --- |
| Source | whole organism | whole organism | whole organism | whole organism | whole organism |
| Details | n=1  Non-symbiotic | n=1  Non-symbiotic | n=1  Unknown symbiosis status | SRX315372  Non-symbiotic | n=1  Unknown symbiosis status |
| Treatment | Control | Control | Control | Control | Control |
| Number of reads | 79,931,258 | 209,875,116 | 205,911,634 | 190,662,106 | 79,893,721 |
| Total assembled base pairs | 110,266,950 (86,578,796) | 139,555,237 (113,152,169) | 110,971,901 (79,668,986) | (80,923,224) | 92,032,807 (71,903,013) |
| Number of transcripts | 146,659 (122,279) | 160,777 (135,489) | 116,120 (88,325) | 93,348 (86,976) | 151,608 (131,812) |
| N10 | 5,228 bp (5101 bp) | 5,740 bp (5,582 bp) | 5,354 bp (5,226 bp) | (6,242 bp) | 3,489 bp (3,220 bp) |
| N30 | 2,703 bp (2530 bp) | 3,141 bp (3,024 bp) | 2,933 bp (2,786 bp) | (3,396 bp) | 1,733 bp (1,458 bp) |
| N50 | 1,516 bp (1358 bp) | 1,862 bp (1,753 bp) | 1,824 bp (1,699 bp) | 2,261 bp (2,063 bp) | 917 bp (727 bp) |
| Maximum length | 35,742 bp | 53,723 bp | 65,337 bp | 37,539 bp | 22,955 bp |
| Average contig length | 751.86 bp (708.04 bp) | 868 bp (835.1 bp) | 955.67 bp (902.00 bp) | 930.41 bp | 607.04 bp (545.50 bp) |
| **CEGMA (before CD-Hit)** |  |  |  |  |  |
| Full length (%) | 95.2 | 96.4 | 97.6 | N/A | 77.4 |
| Full length and partial (%) | 98.0 | 97.6 | 98.8 | N/A | 92.7 |
| **CEGMA (after CD-Hit)** |  |  |  |  |  |
| Full length (%) | 95.6 | 96.4 | 97.6 | 98.0 | 77.4 |
| Full length and partial (%) | 98.4 | 97.6 | 98.8 | 98.8 | 92.7 |

**Table S3. BUSCO results.**

| **Species** | **BUSCO results** |
| --- | --- |
| *A. tenebrosa* (1) | C:87%[D:23%],F:3.9%,M:8.3%,n:429 |
| *A. tenebrosa* (2) | C:88%[D:21%],F:3.0%,M:8.1%,n:429 |
| *A. tenebrosa* (3) | C:89%[D:20%],F:2.5%,M:8.1%,n:429 |
| *A. tenebrosa* (4) | C:89%[D:22%],F:2.0%,M:8.3%,n:429 |
| *A. pallida* | C:88%[D:29%],F:2.7%,M:8.6%,n:429 |
| *A. buddemeieri* | C:80%[D:23%],F:8.1%,M:11%,n:429 |
| *A. elegantissima* | C:93%[D:65%],F:1.3%,M:4.8%,n:429 |
| *A. veratra* | C:87%[D:22%],F:4.1%,M:8.3%,n:429 |
| *C. polypus* (1) | C:86%[D:20%],F:3.2%,M:10%,n:429 |
| *C. polypus* (2) | C:88%[D:30%],F:2.3%,M:9.3%,n:429 |
| *N. annamensis* | C:89%[D:24%],F:1.8%,M:9.0%,n:429 |
| *N. vectensis* | C:89%[D:27%],F:1.8%,M:8.8%,n:429 |
| *Telmatactis* sp. | C:60%[D:12%],F:26%,M:13%,n:429 |

Where C: Complete Single-copy BUSCOs; D: Complete Duplicated BUSCOs; F: Fragmented BUSCOs; M: Missing BUSCOs and n: Total BUSCO groups searched

**Table S4. Number of transcripts in each assembly that received Gene Ontology annotation through BLAST analysis.** Data obtained from Trinotate annotation reports.

| **Species** | **Number of transcripts** |
| --- | --- |
| *A. tenebrosa* (1) | 27,172 |
| *A. tenebrosa* (2) | 24,968 |
| *A. tenebrosa* (3) | 23,348 |
| *A. tenebrosa* (4) | 22,733 |
| *A. pallida* | 29,995 |
| *A. buddemeieri* | 33,653 |
| *A. elegantissima* | 43,791 |
| *A. veratra* | 28,307 |
| *C. polypus* (1) | 25,056 |
| *C. polypus* (2) | 28,970 |
| *N. annamensis* | 22,631 |
| *N. vectensis* | 21,750 |
| *Telmatactis* sp. | 26,594 |


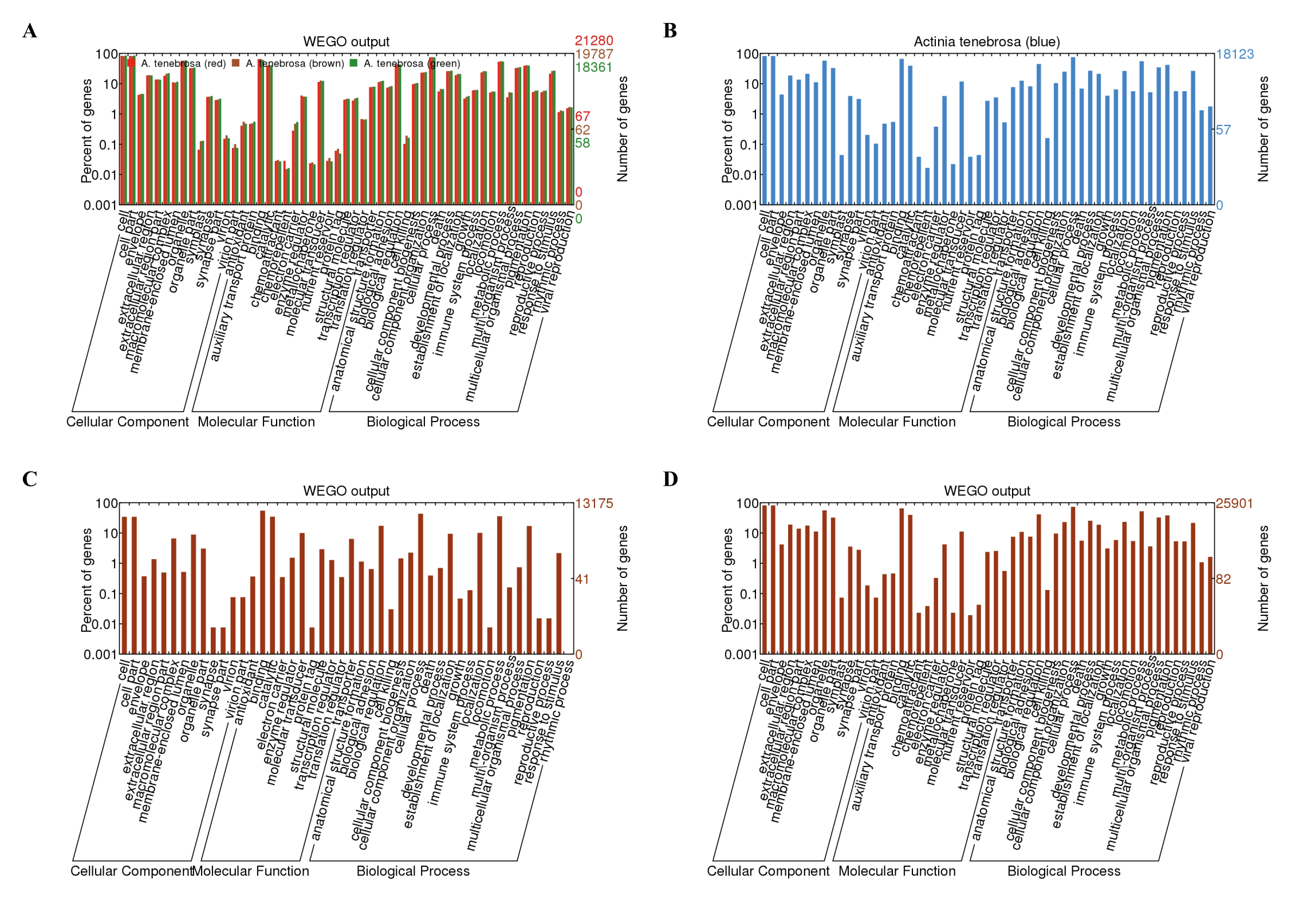


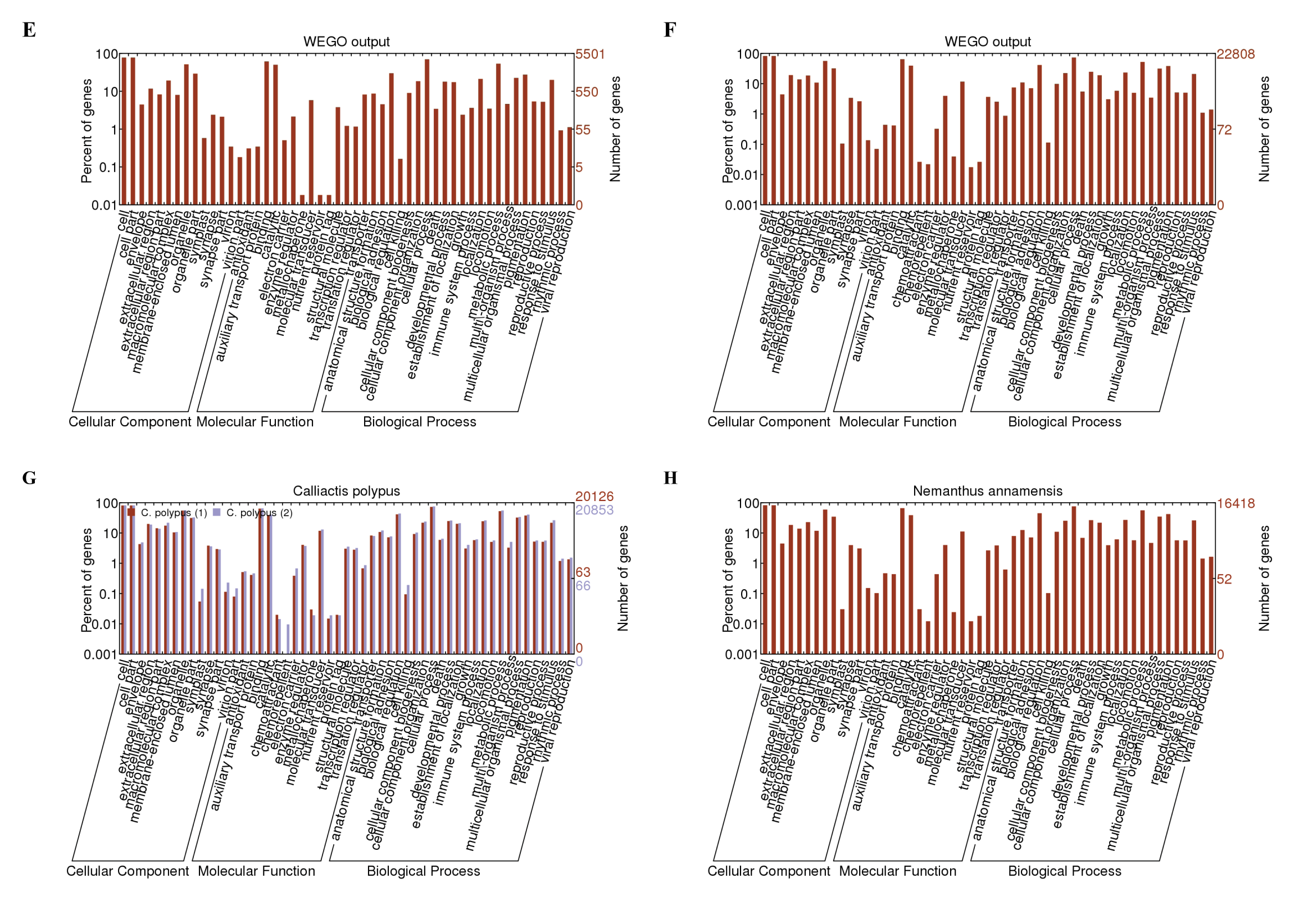


**
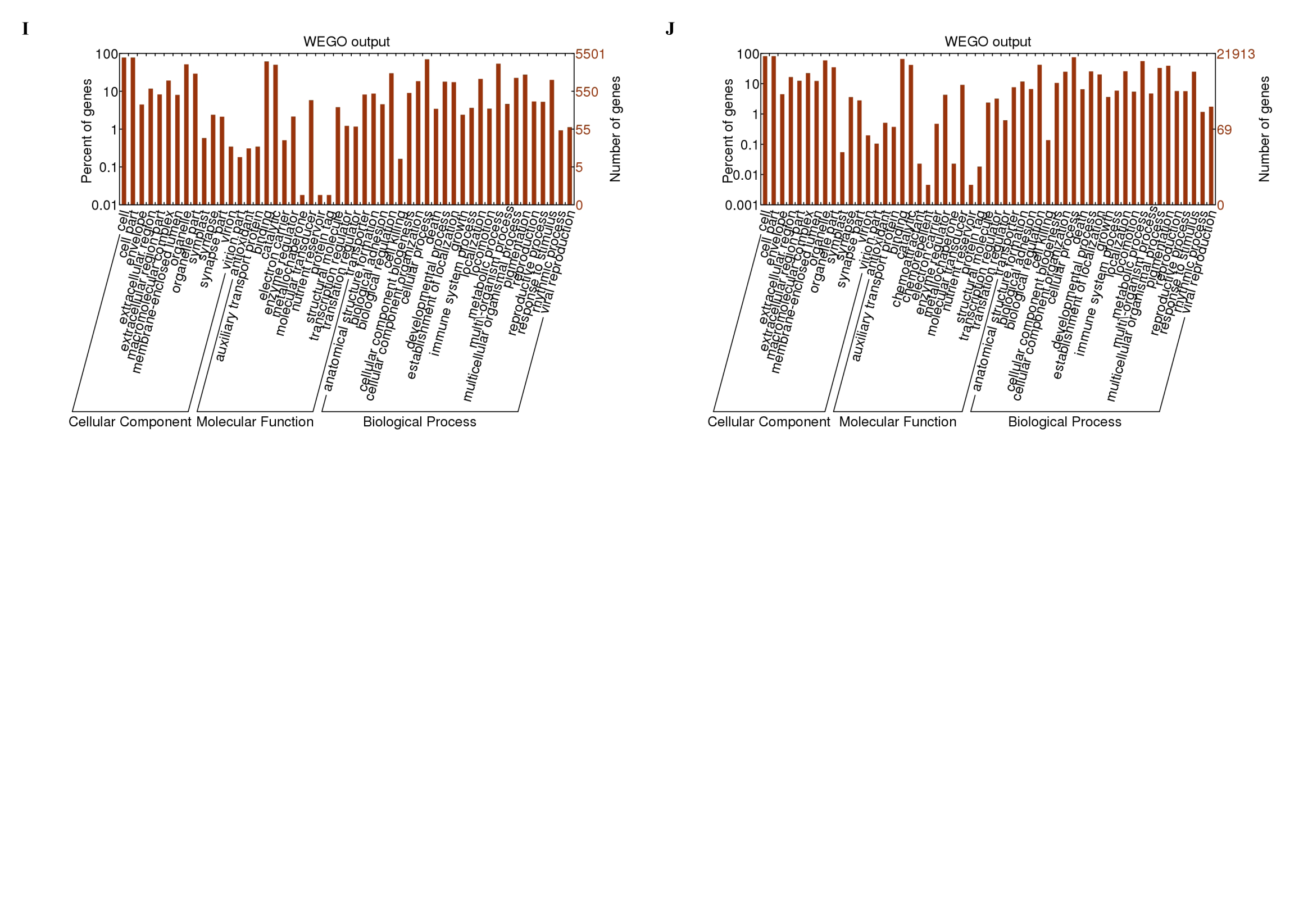
**

**Figure S1. WEGO plots.** Distribution of gene ontology terms shown across three main GO categories (Cellular Component, Molecular Function, Biological Process). All counts refer to number of genes, not transcripts. **(A)** *Actinia tenebrosa* (1=red colourmorph, 2=brown, 3=green); **(B)** *Actinia tenebrosa* (4=blue); **(C)** *Aiptasia pallida*; **(D)** *Anthopleura buddemeieri*; **(E)** *Anthopleura elegantissima*; **(F)** *Aulactinia veratra*; **(G)** *Calliactis polypus* (1, 2); **(H)** *Nemanthus annamensis*; **(I)** *Nematostella* vectensis; **(J)** *Telmatactis* sp.

**
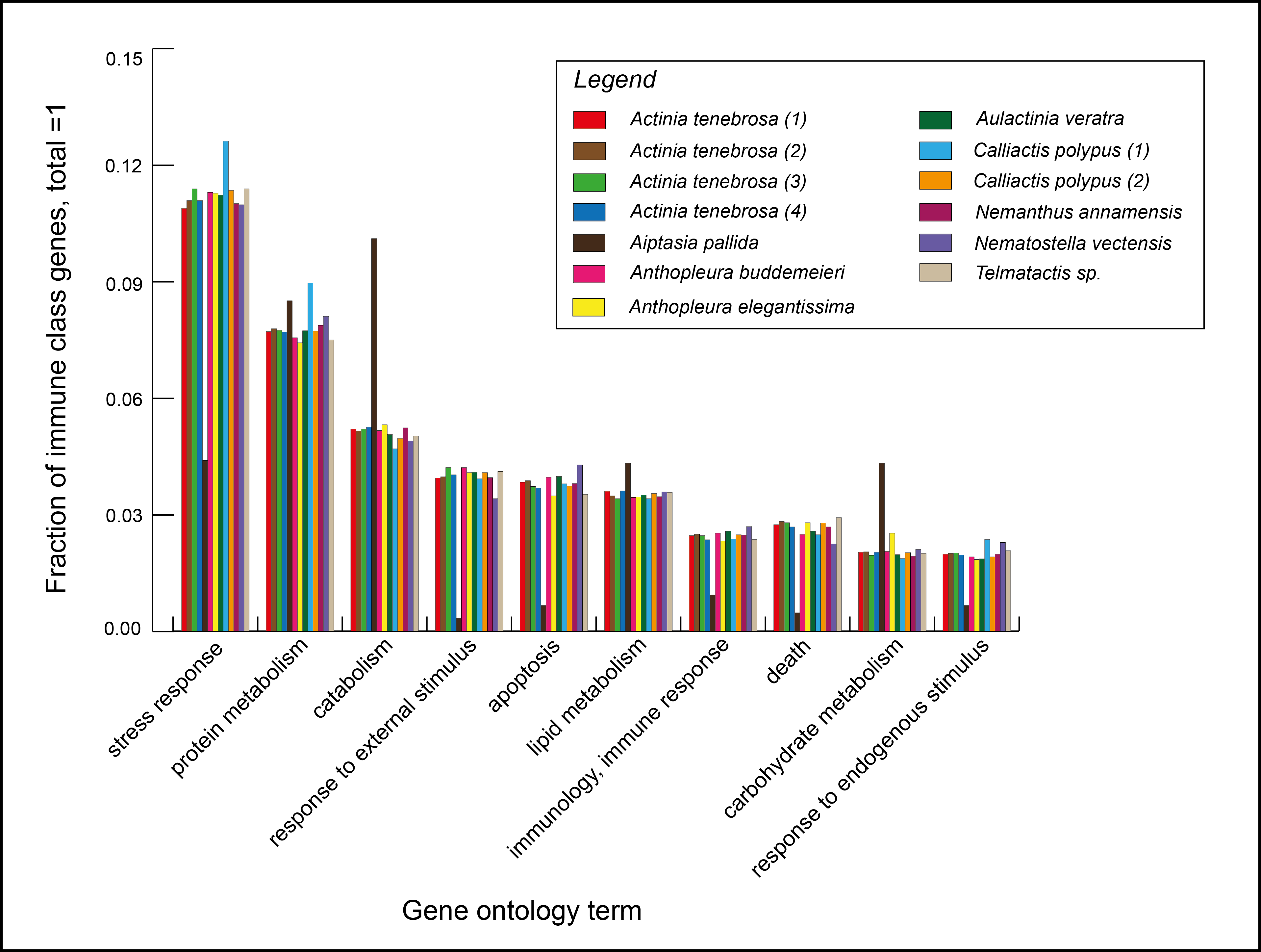
**

**Figure S2. Top 10 immune class Gene Ontology (GO) terms as determined by CateGOrizer for each transcriptome.** Number of genes in each GO category is shown as a fraction of the total genes that are associated with the immune class. ‘Metabolism’ (top hit) is omitted for visualisation purposes.

**Table S5. Top 20 immune class results from CateGOrizer.** Table shows fraction of total ‘immune class’ assigned genes in each GO class (where 1 = total). Full CateGOrizer results are not shown (only top 20) and metabolism (top term in all transcriptomes) is omitted. Total ‘immune class’ gene counts (from full results) are shown at the bottom of the table. Table continues over the next two pages.

| **GO Class ID** | **Definitions** | **Actinia tenebrosa (1)** | ***Actinia tenebrosa* (2)** | ***Actinia tenebrosa* (3)** | ***Actinia tenebrosa* (4)** |
| --- | --- | --- | --- | --- | --- |
| GO:0006950 | stress response | 0.1088 | 0.1108 | 0.1138 | 0.1108 |
| GO:0019538 | protein metabolism | 0.0771 | 0.0778 | 0.0774 | 0.0770 |
| GO:0009056 | catabolism | 0.0520 | 0.0515 | 0.0520 | 0.0525 |
| GO:0009605 | response to external stimulus | 0.0394 | 0.0397 | 0.0421 | 0.0402 |
| GO:0006915 | apoptosis | 0.0383 | 0.0387 | 0.0372 | 0.0368 |
| GO:0006629 | lipid metabolism | 0.0360 | 0.0348 | 0.0341 | 0.0361 |
| GO:0016265 | death | 0.0246 | 0.0249 | 0.0246 | 0.0235 |
| GO:0006955 | immunology, immune response | 0.0274 | 0.0282 | 0.0279 | 0.0268 |
| GO:0005975 | carbohydrate metabolism | 0.0203 | 0.0204 | 0.0195 | 0.0203 |
| GO:0009719 | response to endogenous stimulus | 0.0198 | 0.0200 | 0.0201 | 0.0196 |
| GO:0007155 | cell adhesion | 0.0168 | 0.0164 | 0.0159 | 0.0168 |
| GO:0001816 | cytokine production | 0.0170 | 0.0169 | 0.0166 | 0.0165 |
| GO:0046649 | lymphocyte activation | 0.0158 | 0.0152 | 0.0154 | 0.0154 |
| GO:0009607 | response to biotic stimulus | 0.0141 | 0.0151 | 0.0154 | 0.0153 |
| GO:0042981 | regulation of apoptosis | 0.0140 | 0.0142 | 0.0134 | 0.0135 |
| GO:0009628 | response to abiotic stimulus | 0.0141 | 0.0141 | 0.0148 | 0.0143 |
| GO:0042110 | T cell activation | 0.0090 | 0.0089 | 0.0087 | 0.0091 |
| GO:0016032 | viral life cycle | 0.0073 | 0.0092 | 0.0075 | 0.0077 |
| GO:0030098 | lymphocyte differentiation | 0.0081 | 0.0078 | 0.0080 | 0.0079 |
| GO:0051249 | regulation of lymphocyte activation | 0.0089 | 0.0084 | 0.0087 | 0.0085 |
|  | **Total genes** | **10,957** | **11,006** | **10,914** | **10,828** |

| **GO Class ID** | **Definitions** | ***Aiptasia pallida*** | ***Anthopleura buddemeieri*** | ***Anthopleura elegantissima*** | ***Aulactinia veratra*** |
| --- | --- | --- | --- | --- | --- |
| GO:0006950 | stress response | 0.0439 | 0.1129 | 0.1127 | 0.1122 |
| GO:0019538 | protein metabolism | 0.0850 | 0.0755 | 0.0742 | 0.0773 |
| GO:0009056 | catabolism | 0.1010 | 0.0516 | 0.0531 | 0.0506 |
| GO:0009605 | response to external stimulus | 0.0033 | 0.0421 | 0.0408 | 0.0409 |
| GO:0006915 | apoptosis | 0.0066 | 0.0396 | 0.0348 | 0.0398 |
| GO:0006629 | lipid metabolism | 0.0432 | 0.0344 | 0.0345 | 0.0350 |
| GO:0016265 | death | 0.0093 | 0.0252 | 0.0232 | 0.0257 |
| GO:0006955 | immunology, immune response | 0.0047 | 0.0249 | 0.0279 | 0.0257 |
| GO:0005975 | carbohydrate metabolism | 0.0432 | 0.0205 | 0.0252 | 0.0197 |
| GO:0009719 | response to endogenous stimulus | 0.0066 | 0.0191 | 0.0184 | 0.0186 |
| GO:0007155 | cell adhesion | 0.0040 | 0.0167 | 0.0149 | 0.0165 |
| GO:0001816 | cytokine production | 0.0027 | 0.0161 | 0.0156 | 0.0155 |
| GO:0046649 | lymphocyte activation | 0.0000 | 0.0155 | 0.0139 | 0.0151 |
| GO:0009607 | response to biotic stimulus | 0.0007 | 0.0153 | 0.0153 | 0.0148 |
| GO:0042981 | regulation of apoptosis | 0.0020 | 0.0148 | 0.0130 | 0.0148 |
| GO:0009628 | response to abiotic stimulus | 0.0047 | 0.0143 | 0.0165 | 0.0155 |
| GO:0042110 | T cell activation | 0.0000 | 0.0090 | 0.0081 | 0.0089 |
| GO:0016032 | viral life cycle | 0.0013 | 0.0085 | 0.0077 | 0.0080 |
| GO:0030098 | lymphocyte differentiation | 0.0000 | 0.0083 | 0.0072 | 0.0079 |
| GO:0051249 | regulation of lymphocyte activation | 0.0000 | 0.0079 | 0.0076 | 0.0081 |
|  | **Total genes** | **1,505** | **11,174** | **12,515** | **11,047** |

| **GO Class ID** | **Definitions** | ***Calliactis polypus (1)*** | ***Calliactis polypus* (2)** | ***Nemanthus annamensis*** | ***Nematostella vectensis*** | ***Telmatactis* sp.** |
| --- | --- | --- | --- | --- | --- | --- |
| GO:0006950 | stress response | 0.1261 | 0.1134 | 0.1100 | 0.1097 | 0.1138 |
| GO:0019538 | protein metabolism | 0.0896 | 0.0772 | 0.0787 | 0.0810 | 0.0749 |
| GO:0009056 | catabolism | 0.0469 | 0.0496 | 0.0523 | 0.0489 | 0.0502 |
| GO:0009605 | response to external stimulus | 0.0392 | 0.0408 | 0.0395 | 0.0341 | 0.0411 |
| GO:0006915 | apoptosis | 0.0379 | 0.0373 | 0.0380 | 0.0428 | 0.0352 |
| GO:0006629 | lipid metabolism | 0.0341 | 0.0354 | 0.0346 | 0.0358 | 0.0357 |
| GO:0016265 | death | 0.0237 | 0.0248 | 0.0247 | 0.0269 | 0.0236 |
| GO:0006955 | immunology, immune response | 0.0248 | 0.0278 | 0.0268 | 0.0224 | 0.0292 |
| GO:0005975 | carbohydrate metabolism | 0.0187 | 0.0202 | 0.0193 | 0.0210 | 0.0200 |
| GO:0009719 | response to endogenous stimulus | 0.0236 | 0.0191 | 0.0198 | 0.0228 | 0.0207 |
| GO:0007155 | cell adhesion | 0.0155 | 0.0164 | 0.0173 | 0.0177 | 0.0168 |
| GO:0001816 | cytokine production | 0.0162 | 0.0184 | 0.0171 | 0.0160 | 0.0178 |
| GO:0046649 | lymphocyte activation | 0.0125 | 0.0156 | 0.0155 | 0.0126 | 0.0158 |
| GO:0009607 | response to biotic stimulus | 0.0117 | 0.0163 | 0.0146 | 0.0080 | 0.0168 |
| GO:0042981 | regulation of apoptosis | 0.0125 | 0.0136 | 0.0138 | 0.0152 | 0.0125 |
| GO:0009628 | response to abiotic stimulus | 0.0170 | 0.0134 | 0.0135 | 0.0144 | 0.0138 |
| GO:0042110 | T cell activation | 0.0068 | 0.0092 | 0.0094 | 0.0076 | 0.0093 |
| GO:0016032 | viral life cycle | 0.0068 | 0.0078 | 0.0077 | 0.0072 | 0.0074 |
| GO:0030098 | lymphocyte differentiation | 0.0066 | 0.0081 | 0.0080 | 0.0072 | 0.0083 |
| GO:0051249 | regulation of lymphocyte activation | 0.0065 | 0.0084 | 0.0084 | 0.0077 | 0.0089 |
|  | **Total** | **7,547** | **11,249** | **10,568** | **7,239** | **11,182** |
