# Supplementary material for: Insights into the innate immunome of actiniarians using a comparative genomic approach
Source: BMC Genomics. 2016 Nov 2;17:850. doi: 10.1186/s12864-016-3204-2 (PMC5094078; doi:10.1186/s12864-016-3204-2)
Supplement: Additional file 2: — Identification and characterisation of candidate and novel innate immune genes. Table S6–S10. Counts of number of complete and partial candidate and novel innate immune genes, novel gene BLAST hits, domain architectures and database queries. (DOCX 46 kb) [file 12864_2016_3204_MOESM2_ESM.docx]

# Additional File 2 | Identification and characterisation of candidate and novel innate immune genes

**Insights into the innate immunome of actiniarians using a comparative genomic approach**

Chloe A. van der Burg^1,2^, Peter J. Prentis^3,4^ Joachim M. Surm^1,2^ and Ana Pavasovic^1,2^

^1^School of Biomedical Sciences, Faculty of Health, Queensland University of Technology, GPO Box 2434, Brisbane, Qld 4000

^2^Institute of Health and Biomedical Innovation, Queensland University of Technology, GPO Box 2434, Brisbane, Qld 4000

^3^School of Earth, Environmental and Biological Sciences, Science and Engineering Faculty, Queensland University of Technology, GPO Box 2434, Brisbane, Qld 4000

^4^Institute of Future Environments, Queensland University of Technology, GPO Box 2434, Brisbane, Qld 4000

Corresponding author: Chloe van der Burg.

Email: chloe.vanderburg@hdr.qut.edu.au

## Identification of candidate genes

**Table S6. TLR, MyD88 and NF-κB gene and isoform counts. S**ee Table S6 for *NLR* and *IL-1R* counts. No partial genes found. I, Isoform; G, gene.

| **Species** | ***TLR*** | | ***MyD88*** | | ***NF-κB*** | |
| --- | --- | --- | --- | --- | --- | --- |
|  | I | G | I | G | I | G |
| *A. tenebrosa* (1) | 1 | 1 | 1 | 1 | 2 | 1 |
| *A. tenebrosa* (2) | 1 | 1 | 1 | 1 | 2 | 1 |
| *A. tenebrosa* (3) | 1 | 1 | 1 | 1 | 2 | 1 |
| *A. tenebrosa* (4) | 1 | 1 | 1 | 1 | 2 | 1 |
| *A. pallida* | 0 | 0 | 2 | 1 | 0 | 0 |
| *A. buddemeieri* | 1 | 1 | 2 | 1 | 1 | 1 |
| *A. elegantissima* | 1 | 1 | 1 | 1 | 1 | 1 |
| *A. veratra* | 1 | 1 | 1 | 1 | 1 | 1 |
| *C. polypus* (1) | 0 | 0 | 1 | 1 | 2 | 1 |
| *C. polypus* (2) | 1 | 1 | 1 | 1 | 1 | 1 |
| *N. annamensis* | 1 | 1 | 2 | 1 | 1 | 1 |
| *N. vectensis* | 1 | 1 | 1 | 1 | 0 | 0 |
| *Telmatactis* sp. | 0 | 0 | 0 | 0 | 1 | 1 |

**Table S7. NLR and IL-1R-like gene and partial gene counts.** Table shows number of partial genes found, not number of isoforms. Partial sequences may be missing a start codon (5’ partial), stop codon (3’ partial) or both (internal) and are identified based on the protein domains present.

| **Species** | ***NLR* complete** | ***NLR* partials** | ***NLR* total** | ***IL-1R-*like complete** | ***IL-1R*-like partials** | ***IL-1R*-like total** |
| --- | --- | --- | --- | --- | --- | --- |
| *A. tenebrosa* (1) | 2 | 2 | **4** | 3 | 1 | **4** |
| *A. tenebrosa* (2) | 2 | 7 | **9** | 7 | 0 | **7** |
| *A. tenebrosa* (3) | 2 | 6 | **8** | 4 | 1 | **5** |
| *A. tenebrosa* (4) | 4 | 4 | **8** | 3 | 0 | **3** |
| *A. pallida* | 2 | 8 | **10** | 2 | 1 | **3** |
| *A. buddemeieri* | 3 | 4 | **7** | 2 | 2 | **4** |
| *A. elegantissima* | 0 | 3 | **3** | 3 | 0 | **3** |
| *A. veratra* | 2 | 4 | **6** | 4 | 1 | **5** |
| *C. polypus* (1) | 2 | 3 | **5** | 3 | 2 | **5** |
| *C. polypus* (2) | 4 | 1 | **5** | 5 | 1 | **6** |
| *N. annamensis* | 2 | 2 | **4** | 4 | 0 | **4** |
| *N. vectensis* | 3 | 5 | **8** | 2 | 1 | **3** |
| *Telmatactis* sp. | 0 | 3 | **3** | 1 | 3 | **4** |

## Identification of novel genes

**Table** **S8. TIR-containing gene and isoform counts.**  Counts refer to number of contigs annotated by Pfam with at least one TIR (PF01582) or TIR_2 domain (PF13676).

| **Species** | **Novel TIR/TIR_2-containing** | | **TIR/TIR_2-only** | | **All TIR/TIR_2-containing** | |
| --- | --- | --- | --- | --- | --- | --- |
|  | I | G | I | G | I | G |
| *A. tenebrosa* (1) | 2 | 2 | 13 | 11 | 21 | 20 |
| *A. tenebrosa* (2) | 4 | 4 | 10 | 9 | 22 | 18 |
| *A. tenebrosa* (3) | 3 | 3 | 13 | 10 | 24 | 19 |
| *A. tenebrosa* (4) | 4 | 4 | 13 | 9 | 22 | 18 |
| *A. pallida* | 5 | 5 | 21 | 12 | 31 | 21 |
| *A. buddemeieri* | 5 | 5 | 20 | 16 | 33 | 25 |
| *A. elegantissima* | 4 | 4 | 9 | 7 | 19 | 15 |
| *A. veratra* | 4 | 3 | 14 | 12 | 28 | 19 |
| *C. polypus* (1) | 5 | 5 | 17 | 13 | 30 | 23 |
| *C. polypus* (2) | 5 | 5 | 11 | 10 | 29 | 23 |
| *N. annamensis* | 7 | 6 | 12 | 8 | 30 | 20 |
| *N. vectensis* | 6 | 6 | 12 | 6 | 25 | 16 |
| *Telmatactis* sp. | 2 | 2 | 12 | 10 | 19 | 16 |

**Table S9. Novel TIR-containing genes.** All contigs shown include a Toll/Interleukin-1 receptor homology domain (either TIR (PF01582) or TIR_2 (PF13676)) as part of their architecture. Partial contigs are also shown; these are denoted with an asterisk next to the contig ID and the missing domain is listed in brackets under Pfam domains. All BLASTx and BLASTp hits are shown as the UniProtKB ID for each contig. Pfam domains found include the following: LRR, leucine-rich repeat (CL0022); Miro, Miro-like protein (PF08477-Roc); Ras, Ras family (PF00071); BTK, Bruton's tyrosine kinase-type zinc-finger motif (PF00779); CBM, carbohydrate binding motif (CL0535); SAM, Sterile alpha motif (CL0003); Arm, Armadillo (PF00514); DUF1863, MTH538 TIR-like domain (PF08937); Pkinase_Tyr, protein tyrosine kinase (PF07714); Pkinase, Protein kinase (PF00069); Death domain (PF00531); SH3_2, Src homology 3 (PF07653); V-ATPase_H_N, Subunit H of the V-TYPE ATPase of *Saccharomyces cerevisiae* PF03224)*.* Species shown include: *Actinia tenebrosa*, *Anthopleura buddemeieri, Anthopleura elegantissima*, *Aiptasia pallida*, *Aulactinia veratra*, *Calliactis polypus*, *Nematostella vectensis* and *Telmatactis* sp..

| **Species** | **Contig** | **Pfam domains** | **Swiss-Prot BLASTx and/or BLASTp hit** | **TrEMBL BLASTx and/or BLASTp hit** |
| --- | --- | --- | --- | --- |
| *A. tenebrosa* (1) | c71481_g1_i4 | LRR, Miro, Ras | LRRK2_MOUSE  GBPC_DICDI | A7T2M0_NEMVE |
|  | c67223_g1_i1 | SAM | none | A8DUR5_NEMVE |
| *A. tenebrosa* (2) | c67726_g1_i1 | BTK | none | A7ST59_NEMVE |
|  | c64000_g1_i2 | CBM | XYNA_THESA | D1LX78_SACKO |
|  | *c66001_g1_i1 | LRR, Miro, Ras (no TIR) | FBXL2_PONAB  FBXL2_HUMAN | A7T2M0_NEMVE |
|  | c27393_g1_i1 | SAM | none | A8DUR5_NEMVE |
| *A. tenebrosa* (3) | c51068_g1_i1 | BTK | none | A7ST59_NEMVE |
|  | c49904_g1_i3 | LRR, Miro, Ras | FBXL2_HUMAN  FBXL2_PONAB | A7T2M0_NEMVE |
|  | c16023_g1_i1 | SAM | none | A8DUR5_NEMVE |
| *A. tenebrosa* (4) | TR26124\|c2_g1_i3 | LRR, Miro, Ras | FBXL2_PONAB  FBXL2_HUMAN | A7T2M0_NEMVE |
|  | TR2621\|c0_g1_i1 | CBM | XYNA_THESA | K1QWV6_CRAGI  D1LX78_SACKO |
|  | TR12895\|c0_g1_i1 | BTK | none | A7ST59_NEMVE |
|  | TR24546\|c0_g1_i1 | SAM | none | A8DUR5_NEMVE |
| *A. pallida* | c75576_g1_i2 | BTK | none | A7ST59_NEMVE |
|  | c72098_g1_i1 | LRR, Miro, Ras | FBXL2_BOVIN  FBXL2_PONAB | A7T2M0_NEMVE |
|  | c79565_g1_i3 | Pkinase_Tyr, Pkinase, Death | PATS1_DICDI | A7RN39_NEMVE |
|  | c71016_g1_i1 | SAM | none | A8DUR5_NEMVE |
|  | c70774_g1_i2 | Arm | none | A7RU69_NEMVE |
| *A. buddemeieri* | c121220_g1_i1 | DUF1863 | none | F2UPF3_SALR5 |
|  | c117321_g2_i4 | DUF1863 | none | F2UPF3_SALR5 |
|  | *c120512_g3_i1 | Miro, Ras (no LRR) | LRRK2_HUMAN | A7T2M0_NEMVE |
|  | c114941_g1_i4 | CBM | XYNA_THESA | A7RN39_NEMVE  D1LX78_SACKO |
|  | c115904_g1_i1 | SAM | none | A8DUR5_NEMVE |
| *A. elegantissima* | c16802_g1_i1 | BTK | none | A7ST59_NEMVE |
|  | c46533_g1_i1 | LRR, Miro, Ras | FBXL2_PONAB  FBXL2_HUMAN | A7T2M0_NEMVE |
|  | c56670_g1_i4 | DUF1863 | TLR13_MOUSE | A7SFW6_NEMVE |
|  | c48043_g1_i1 | SAM | none | A8DUR5_NEMVE |
| *A. veratra* | c93217_g1_i1 | LRR, Miro, Ras | FXL20_MOUSE | A7T2M0_NEMVE |
|  | c92865_g2_i1 | CBM | XYNA_THESA | K0J6V0_AMPXN  D1LX78_SACKO |
|  | c92865_g2_i2 | CBM | XYNA_THESA | A7RN39_NEMVE  W4Z3K9_STRPU |
|  | c90880_g1_i1 | SAM | none | A8DUR5_NEMVE |
| *C. polypus* (1) | c64988_g1_i1 | LRR, Miro, Ras | FBXL7_MOUSE  FXL20_MOUSE | A7T2M0_NEMVE |
|  | c66041_g3_i2 | DUF1863 | TOLL_DROME | A7RRY9_NEMVE |
|  | c62111_g2_i5 | DUF1863 | TLR2_CANFA  TLR22_CHICK | A7SFW6_NEMVE |
|  | *c66411_g1_i2 | SH3, Death (no Pkinase) | GBPC_DICDI | A7RN39_NEMVE |
|  | c57233_g1_i1 | SAM | none | A8DUR5_NEMVE |
| *C. polypus* (2) | c37075_g1_i1 | BTK | none | A7ST59_NEMVE |
|  | c67557_g1_i2 | LRR, Miro, Ras | FBXL7_MOUSE  FXL20_MOUSE | A7T2M0_NEMVE |
|  | c70990_g1_i6 | Pkinase_tyr, Pkinase, SH3_2, Death | GBPC_DICDI | A7RN39_NEMVE |
|  | c65740_g1_i1 | V-ATPase_H_N | none | A7RU69_NEMVE |
|  | c64997_g1_i1 | SAM | none | A8DUR5_NEMVE |
| *N. annamensis* | TR16649\|c0_g1_i1 | LRR, Miro, Ras | FBXL2_BOVIN | A7T2M0_NEMVE |
|  | TR22639\|c0_g1_i1 | V-ATPase_H_N | none | A7RU69_NEMVE |
|  | TR24266\|c0_g1_i1 | BTK | none | A7ST59_NEMVE |
|  | TR28129\|c3_g2_i3 | V-ATPase_H_N | none | A7RU69_NEMVE |
|  | TR49481\|c0_g1_i1 | SAM | none | A8DUR5_NEMVE |
|  | TR28359\|c3_g1_i3 | DUF1863 | none | F2UPF3_SALR5 |
|  | TR28359\|c3_g1_i6 | DUF1863 | none | F2UPF3_SALR5 |
| *N. vectensis* | c15865_g1_i1 | BTK | none | A7ST59_NEMVE |
|  | c15882_g1_i1 | DUF1863 | none | A9V6F2_MONBE |
|  | c30739_g1_i2 | LRR, Miro, Ras | FXL15_DANRE  FBXL7_HUMAN | A7T2M0_NEMVE |
|  | c32607_g1_i2 | Pkinase_tyr, Pkinase, Death | GBPC_DICDI | A7RN39_NEMVE |
|  | c24663_g1_i1 | SAM | none | A8DUR5_NEMVE |
| *Telmatactis* sp. | *c72038_g1_i7 | Ras, Miro (no LRR) | GBPC_DICDI | A7T2M0_NEMVE |
|  | c88797_g1_i1 | SAM | none | A8DUR5_NEMVE |

### Taxonomically-restricted novel genes

**Table S10. Identified proteins with domain architectures similar to the three novel genes.**  Table shows species from other taxa found with the domain combinations similar to Novel Genes 1, 2 and 3, which may be potentially related architectures. Domains shown: LRR, Leucine-rich repeat (any kind from clan CL0022); Roc, Ras of complex (PF08477); COR, C-terminal of Roc (PF16095); TIR_2, Toll/interleukin-1 receptor homology domain (PF13676); MBT, malignant brain tumour (PF02820); repeat, unknown function); C2, Ca2+-dependent membrane-targeting module (PF00168); BTK, Bruton's tyrosine kinase-type zinc-finger motif (PF00779); Pkinase_Tyr, tyrosine protein kinase (PF07714); Neuralized (PF07177). Multiple UniProtKB IDs shown for some species where multiple proteins were found.

| **Pfam domains** | **Species** | **UniProtKB IDs** | |
| --- | --- | --- | --- |
| **Novel Gene 1** |  |  |  |
| LRR, Roc, TIR_2 | *Porphyromonas gingivalis* | Q7MTS7_PORGI |  |
| LRR, Roc, COR, TIR_2 | *Nostoc punctiforme*  *Coleofasciculus chthonoplastes*  *Thiohalocapsa sp.*  *Porphyromonas gingivalis*  *Cyanothece sp.*  *Fibrisoma limi*  *Acaryochloris marina*  *Vibrio nigripulchritudo*  *Leptolyngbya sp*  *Haliscomenbacter hydrossis*  *Thioflavicoccus mobilis*  *Nonlabens dokdonensis*  *Methylomonas methanica* | B2IUT6_NOSP7  B2IXB5_NOSP7  B4VY01_9CYAN  V4J0L0_9GAMM  Q7MTS7_PORGI  U2LIU3_PORGN  B2RLS0_PORG3  U2IN74_PORGN  U2JZW2_PORGN  W1R842_PORGN  B7KEE8_CYAP7  I2GJA4_9BACT | I2GMB1_9BACT  B0CFH1_ACAM1  U4F8Z6_9VIBR  U4ESJ1_9VIBR  U4F3M8_9VIBR  U9VPQ7_9CYAN  F4L2I2_HALH1  F4KSN1_HALH1  L0GT29_9GAMM  L7W2S5_NONDD  G0A2L9_METMM |
| Roc, COR, TIR_2 | *Haliscomenobacter hydrossis*  *Guillardia theta*  *Chondrus crispus*  *Lottia gigantea*  *Flavobacterium cauense* | F4KSK8_HALH1  L1I8G5_GUITH  L1IP31_GUITH  L1JCH1_GUITH  R7QNN8_CHOCR | V4BHZ2_LOTGI  V6S3T0_9FLAO  A8UP62_9FLAO |
| TIR_2, Roc, COR, TIR_2 | *Leptolyngbya sp* | U9W764_9CYAN |  |
| Roc, COR, DEATH, TIR_2 | *Strongylocentrotus purpuratus* | W4ZA34_STRPU |  |
| MBT, LRR_8, Roc, COR, TIR_2 | *Trichoplax adhearens* | B3S268_TRIAD |  |
| C2, LRR, Roc, COR, TIR_2 | *Crassostrea gigas* | K1PB18_CRAGI |  |
| Ras, TIR_2 | *Thermoplasmatales archaeon I-plasma* | T0MU79_9EURY |  |
| **Novel Gene 2** |  |  |  |
| TIR_2, BTK | *Phytophthora ramorum*  *Guillardia theta* | H3H1G6_PHYRM  L1JXD3_GUITH |  |
| **Novel Gene 3** |  |  |  |
| Pkinase_Tyr, Roc, COR, Death, TIR_2 | *Nematostella vectensis* | A7RN39_NEMVE |  |
| Neuralized, Roc, COR, Death, TIR_2 | *Strigamia maritima*  *Crassostrea gigas* | T1IR33_STRMM  K1QWV6_CRAGI |  |
| Roc, COR, Death, TIR_2 | *Strongylocentrotus purpuratus* | W4ZA34_STRPU |  |
| COR, Death, TIR_2 | *Tetranychus urticae* | T1K6Y9_TETUR |  |
