# Supplementary material for: Insights into the innate immunome of actiniarians using a comparative genomic approach
Source: BMC Genomics. 2016 Nov 2;17:850. doi: 10.1186/s12864-016-3204-2 (PMC5094078; doi:10.1186/s12864-016-3204-2)
Supplement: Additional file 3: — PCR validation of candidate and novel genes. Tables S11–S14. Primer lists, PCR protocols and GenBank accession numbers. (DOCX 32 kb) [file 12864_2016_3204_MOESM3_ESM.docx]

# Additional File 3 | PCR validation of candidate and novel genes

**Insights into the innate immunome of actiniarians using a comparative genomic approach**

Chloe A. van der Burg^1,2^, Peter J. Prentis^3,4^ Joachim M. Surm^1,2^ and Ana Pavasovic^1,2^

^1^School of Biomedical Sciences, Faculty of Health, Queensland University of Technology, GPO Box 2434, Brisbane, Qld 4000

^2^Institute of Health and Biomedical Innovation, Queensland University of Technology, GPO Box 2434, Brisbane, Qld 4000

^3^School of Earth, Environmental and Biological Sciences, Science and Engineering Faculty, Queensland University of Technology, GPO Box 2434, Brisbane, Qld 4000

^4^Institute of Future Environments, Queensland University of Technology, GPO Box 2434, Brisbane, Qld 4000

Corresponding author: Chloe van der Burg.

Email: chloe.vanderburg@hdr.qut.edu.au

## Primer list

**Table S11. Primer list.** Primers for amplifying Toll-like receptor (*TLR*), *MyD88* (MyD), *NF-κB* (NFKB), *NLR* (N), *IL-1R* (IL), novel genes (NG) and CniFL from *Actinia tenebrosa* (At), *Anthopleura buddemeieri* (Ab), *Aulactinia veratra* (Av) and *Calliactis polypus* (Cp).

| **Primer name** | | | **Primer sequence 5’ - 3’** | | | **Product size (bp)** | |
| --- | --- | --- | --- | --- | --- | --- | --- |
| **Toll-like receptor primers** | | | | | | | |
| AtTLR_F1 | GATGTCGTCATGTCTGCCCA | | | | | 1250 | |
| AtTLR_R1 | TGCAGCAACAATCCCTTGGA | | | | |  | |
| AtTLR_F2 | AGGAAACGGACTCAAAGCCA | | | | | 1240 | |
| AtTLR_R2 | GGATCGGCAGTCACACTCAA | | | | |  | |
| AtTLR_F3 | GAGCATGCGTGGTTACCTTG | | | | | 1230 | |
| AtTLR_R3 | TGAGTTTGCCATGCCCTGAC | | | | |  | |
| AbTLR_F1 | TTCAGCCGTACGTGATGCTT | | | | | 1264 | |
| AbTLR_R1 | GGTAGAGCTCCGTGTTCAACT | | | | |  | |
| AbTLR_F2 | TGGACTCAAAGCGATTGGGA | | | | | 1175 | |
| AbTLR_R2 | ACTTGCGTGACGTTAGCACT | | | | |  | |
| AbTLR_F3 | CCTCATAGACGCTCGTGACA | | | | | 1244 | |
| AbTLR_R3 | TCAGCCCAGAGGTTGTTTCTT | | | | |  | |
| AvTLR_F1 | GTCAAGAAGTGCACCATGTCG | | | | | 1250 | |
| AvTLR_R1 | CTTCGAAGTCGGTTTCGGCT | | | | |  | |
| AvTLR_F2 | CATTTAAGGACACAACCCGCA | | | | | 1164 | |
| AvTLR_R2 | AGATTGCCTTGCCAGTAGGT | | | | |  | |
| AvTLR_F3 | CCTTGACAACGTGACAGTCCT | | | | | 1091 | |
| AvTLR_R3 | CCACTGTTTCAGCGGCACTA | | | | |  | |
| **MyD88 primers** | | | | | | | |
| AtMyD_F1 | TCCTTCTGACAGGAAGTTGAC | | | | | 1079 | |
| AtMyD_R1 | CAAATCAGTGAGCACTGTCCA | | | | |  | |
| AbMyD_F1 | TGGAATGAGACAGGAAGCTGG | | | | | 1118 | |
| AbMyD_R1 | GGGAAACCAAACCTCACTTGC | | | | |  | |
| AvMyD_F1 | CAAATTCCAAGATGGCGAGCA | | | | | 930 | |
| AvMyD_R1 | CGACTGCGCAGGAGACTAAT | | | | |  | |
| **NF-κB primers** | | | | | | | |
| AtNFKB_F1 | AGAGGAAGCTTGGATGCCTT | | | | | 1018 | |
| ActNFKB_R1 | ACCTGTAACGCAGCCTGAAT | | | | |  | |
| AtNFKB_F2 | AATCTTGCGCTGTCTGTTGC | | | | | 1125 | |
| AtNFKB_R2 | GTTGCCATGACGGTCACAAC | | | | |  | |
| AtNFKB_F3 | CATGAAGGACCTGCCAGGAG | | | | | 983 | |
| AtNFKB_R3 | CGGCTGGTTACTCGATGACA | | | | |  | |
| AbNFKB_F1 | AGAGGAAGCTTGGATGCCTT | | | | | 1106 | |
| AbNFKB_R1 | GCCACTGGCAGGATCCATTT | | | | |  | |
| AbNFKB_F2 | ACCTGACGATACTGGCAACTT | | | | | 1170 | |
| AbNFKB_R2 | TCCCTGTTGCCTCTGATGAC | | | | |  | |
| AbNFKB_F3 | GCCTAATGAAGGGCAATGCG | | | | | 945 | |
| AbNFKB_R3 | TTATTGGCCGGCTGTGATGT | | | | |  | |
| AvNFKB_F1 | TGGCGGACGGTTTGTGTTTA | | | | | 1199 | |
| AvNFKB_R1 | TGGACGTCTGATGGAGCAAA | | | | |  | |
| AvNFKB_F2 | AGGCACCATCGTCTTGTCAG | | | | | 1168 | |
| AvNFKB_R2 | CCATCCTTGGCCTCCACATT | | | | |  | |
| AvNFKB_F3 | CATTGCTTGTGCTCAGGGTG | | | | | 938 | |
| AvNFKB_R3 | CAGCTGGCCGTGACAATCTT | | | | |  | |
| **NLR primers** |  | | | | |  | |
| AtN_F1 | AATGTTGATTCGCGATTGGGC | | | | | 1007 | |
| AtN_R1 | GACCACACTGAAGAAGGCCA | | | | |  | |
| AtN_F2 | GGAGGAAAGTGTGTCGCCAT | | | | | 809 | |
| AtN_R2 | ACAGTTCGCCTAAACAGGGC | | | | |  | |
| AtN_F3 | ACCAGCGATACTGTGATGACG | | | | | 712 | |
| AtN_R3 | CACAAGTGTTCTTTCATCTCGC | | | | |  | |
| AvN_F1 | AGACTTGGAAGACGCTCGTT | | | | | 1132 | |
| AvN_R1 | TTGACAGCACTTGGACGTGT | | | | |  | |
| AvN_F2 | TGGTATGTGGACCTCAGGGT | | | | | 1068 | |
| AvN_R2 | AAACACCTTCATGGCATCGC | | | | |  | |
| AvN_F3 | AAGACCGTCGAACCAACTCC | | | | | 1137 | |
| AvN_R3 | TCCTACCTCGTCCATCAGGTT | | | | |  | |
| AvN_F4 | AACTGTGCTGTCGCTGAAGT | | | | | 1135 | |
| AvN_R4 | CTGCACCGTATACCCGTACC | | | | |  | |
| CpN_F1 | GGTTCTGAACAATTTCACGCC | | | | | 1078 | |
| CpN_R1 | TGAGGCATCTCTTCCCACAT | | | | |  | |
| CpN_F2 | GACCAGGCAAAGGATCCAAGA | | | | | 1193 | |
| CpN_R2 | TTTGGGACAACATTGAACCACT | | | | |  | |
| CpN_F3 | TTGCTACTCTAGCACGGTGT | | | | | 1122 | |
| CpN_R3 | TGTAAACCACTGGGCAACCA | | | | |  | |
| CpN_F4 | TGGGTGGAAGGATGTTCAGC | | | | | 946 | |
| CpN_R4 | ACAGAAAGCACTATTTGTGCCA | | | | |  | |
| **IL-1R primers** |  | | | | |  | |
| Atil_F1 | AGCGAGGACATACGGTGTCT | | | | | 918 | |
| Atil_R1 | CTCAGCATGTAAACGGCTGC | | | | |  | |
| Atil_F2 | TCTTCATACATTGGCGAGGCA | | | | | 1047 | |
| Atil_R2 | AGGGAAATGGAGAACCTCCC | | | | |  | |
| Abil_F1 | CTGACCTGACTGCGGTTTCA | | | | | 1060 | |
| Abil_R1 | TCCTTGATGGACTTGGCAGG | | | | |  | |
| Abil_F2 | CTGTCCATGGAACGACAGCA | | | | | 1046 | |
| Abil_R2 | TACCTGCAAACAGTCACGCA | | | | |  | |
| Avil_F1 | TTTGATCGCGTAACTGTAGCG | | | | | 1101 | |
| Avil_R1 | CCTCCTGAGTAAACGGTGCC | | | | |  | |
| Avil_F2 | CCCACCCAGCTCCATGACTA | | | | | 1080 | |
| Avil_R2 | ACTTAGGCCCTGTCACGGTA | | | | |  | |
| Cpil_F1 | AATGGGTGGCTGATCGTTTG | | | | | 1150 | |
| Cpil_R1 | CCTTCGTCATCCTCAGTGACA | | | | |  | |
| Cpil_F2 | CCGGCAAGCCATCTCTTTCTT | | | | | 1190 | |
| Cpil_R2 | TATCGTCGTCTTCTTGCAGCC | | | | |  | |
| **Novel gene primers** | | | |  | | |  |
| AtNG1_F1 | | AGAGTTACAATGGCTGTGCTT | | | 1000 | | |
| AtNG1_R1 | | TGGGATACCAATCGAGCAGA | | |  | | |
| AtNG1_F2 | | AGCCGTTGCCATAGCTTCAT | | | 1000 | | |
| AtNG1_R2 | | GGGAAAGCCAATACACCACG | | |  | | |
| AtNG1_F3 | | CGTTCGACTCCTGTCAGCTT | | | 684 | | |
| AtNG1_R3 | | TCGAACAAACGTCAAAGGCT | | |  | | |
| AtNG2_F1 | | TGTTAGTCGCGTCGATAGCC | | | 1000 | | |
| AtNG2_R1 | | CGACGGCAAAGCATTATGGG | | |  | | |
| AtNG2_F2 | | GGGCACGTGACCATTTCAAG | | | 977 | | |
| AtNG2_R2 | | GGGACTCATCAGAGAGGCTG | | |  | | |
| AtNG2_F3 | | GTGCACCTTCAACAAGCACC | | | 989 | | |
| AtNG2_R3 | | CCGAAAGTGCATCCAAGGTC | | |  | | |
| AtNG2_F4 | | GCATGCGTTCCAACGACTATC | | | 961 | | |
| AtNG2_R4 | | TCGGGCTATCTGGCTTCAAC | | |  | | |
| AtNG2_F5 | | TGTTATCTTCAGACACCAGCC | | | 871 | | |
| AtNG2_R5 | | ATGAAGTATGGCTTCGTCGT | | |  | | |
| CpNG3_F1 | | ATGCGCCATGCAGTGTGTA | | | 850 | | |
| CpNG3_R1 | | GGACGAAATCGAAGTGGACG | | |  | | |
| CpNG3_F2 | | GGCTAGGCCACCCATTTGTA | | | 870 | | |
| CpNG3_R2 | | ACCGGTGAAACCCGTTACTT | | |  | | |
| CpNG3_F3 | | TGGGACAAAGCCATCCACAA | | | 948 | | |
| CpNG3_R3 | | GTTCGGCAAGGTGCATTTGAT | | |  | | |
| CpNG3_F4 | | CTTCCTGGTCATTTGGCCGT | | | 954 | | |
| CpNG3_R4 | | GCAACTTGTCCACACCCATC | | |  | | |
| CpNG3_F5 | | AGCGATCATTCTTGCGAGGT | | | 962 | | |
| CpNG3_R5 | | TTGGAGGCTGTGTTCCTAGAG | | |  | | |
| CpNG3_F6 | | ATGGAACCACATTAGGGCGG | | | 886 | | |
| CpNG3_R6 | | TGCTGTACAAGGACAAGCTGA | | |  | | |
| **CniFL gene primers** | |  | | |  | | |
| AtCniFL1_F1 | | AAAGCATCGGCCATCTCTCA | | | 938 | | |
| AtCniFL1_R1 | | GGAATCACCAGCCTACGGTT | | |  | | |
| AtCniFL1_F2 | | AGGCTTGACTGTATCATCACCT | | | 952 | | |
| AtCniFL1_R2 | | CACTCACTCACACCCTACTCG | | |  | | |
| AtCniFL2_F1 | | CCATCGTTTGGTCGTGTTCG | | | 1018 | | |
| AtCniFL2_R1 | | GTTTGGGATGTCCGATTGCG | | |  | | |
| AtCniFL2_F2 | | GCTGGAGTAAAGTGGGAGGT | | | 990 | | |
| AtCniFL2_R2 | | AATCCACACGTAGCACCGAG | | |  | | |
| AtCniFL2_F3 | | TTTGCACAGCAACAAGTCCG | | | 903 | | |
| AtCniFL2_R3 | | TAGAAACACCGACGCAGCTC | | |  | | |
| AbCniFL1_F1 | | CTAAACGCTACGTGCTTGCC | | | 1011 | | |
| AbCniFL1_R1 | | GCCAGTGACGTGACATGTTG | | |  | | |
| AbCniFL1_F2 | | GACCAGGTCGAAGAGGCAAA | | | 1133 | | |
| AbCniFL1_R2 | | CTTGGCTGTCTGAAGGTCGT | | |  | | |
| AbCniFL1_F3 | | TCACCCTGAAACCAGCCTTG | | | 1046 | | |
| AbCniFL1_R3 | | TCATGGTAAGTGGCAATGGGT | | |  | | |
| AbCniFL2_F1 | | GACTGTCGGTCTGCCTGTTT | | | 937 | | |
| AbCniFL2_R1 | | CTTTGGGTCCCATCATGCCT | | |  | | |
| AbCniFL2_F2 | | ATGGCAAAGTCGTTCGTGGA | | | 1121 | | |
| AbCniFL2_R2 | | GGTTCCTTGACTGTCGGGTT | | |  | | |
| AbCniFL2_F3 | | TGGTACAAGGACCTCCCGAT | | | 944 | | |
| AbCniFL2_R3 | | CCTTAGACATGCATTGCGGC | | |  | | |

## PCR Protocols

**Table S12. PCR protocols used for initial sequence validation.** Amount of MgCl_2_ and cDNA increased for PCRs where no PCR products were initially obtained.

| **Reagent** | **Amount (μL)** |
| --- | --- |
| ddH_2_O | 16 |
| 5 x MyFi buffer | 5 |
| F primer (10μM) | 1 |
| R primer (10μM) | 1 |
| MgCl_2_ | 0 |
| MyFi DNA polymerase | 1 |
| cDNA | 1 |
| **TOTAL** | **25** |

**Table S13. Thermocycler conditions used for initial sequence validation.** Amount of cycles increased to 35 for PCRs where only low amounts of PCR product were obtained.

| **Temperature (°C)** | **Time** | **Cycles** |
| --- | --- | --- |
| 95 | 1 min |  |
| 95 | 15 sec | x 30 |
| 52 | 15 sec | x 30 |
| 72 | 30 sec | x 30 |
| 72 | 5 min |  |
| 10 | hold |  |

## Genbank

**Table S14. NCBI GenBank® accession numbers.** Genes validated through PCR amplification of the open reading frame (ORF). Novel gene 1 domains: TIR_2 (x2) and BTK motif. Novel gene 2 domains: LRR, small GTPase and TIR_2. Novel gene 3 domains: Pkinase, SH3, Roc, COR, Death, TIR.

| **Species** | **Gene** | **Length of ORF (nucleotides)** | **GenBank® accession number** |
| --- | --- | --- | --- |
| *A. tenebrosa* | *TLR* | 2982 | KT751519 |
|  | *MyD88* | 834 | KT792961 |
|  | *NF-κB* | 2397 | KT792966 |
|  | *IL-1R* | 1662 | KU710366 |
|  | *NLR* | 1866 | KU710365 |
|  | Novel gene 1 | 2031 | KU710363 |
|  | Novel gene 2 | 3828 | KU710364 |
|  | CniFL1 | 1560 | KX592607 |
|  | CniFL2 internal gap | 2076‡ | KX592608 |
| *A. buddemeieri* | *TLR* | 2949 | KT792959 |
|  | *MyD88* | 837 | KT792962 |
|  | *NF-κB* | 2592 | KT792965 |
|  | *IL-1R* 3’ partial | 765* | KX223878 |
|  | CniFL1 | 2121 | KX592609 |
|  | CniFL2 | 2190 | KX592610 |
| *A. veratra* | *TLR* | 2904 | KT792960 |
|  | *MyD88* | 810 | KT792963 |
|  | *NF-κB* | 2685 | KT792964 |
|  | *NLR* internal gap | 3243‡ | KX223880 |
| *C. polypus* | *IL-1R* | 1095 | KU710367 |
|  | *NLR* 5’ partial | 1299* | KX223879 |
|  | Novel gene 3 | 4017 | KU710368 |

*Length of submitted sequence for partial sequences. ‡Approximate length of ORF.
